# DNMT1 reads heterochromatic H4K20me3 to reinforce LINE-1 DNA methylation

**DOI:** 10.1038/s41467-021-22665-4

**Published:** 2021-05-03

**Authors:** Wendan Ren, Huitao Fan, Sara A. Grimm, Jae Jin Kim, Linhui Li, Yiran Guo, Christopher James Petell, Xiao-Feng Tan, Zhi-Min Zhang, John P. Coan, Jiekai Yin, Dae In Kim, Linfeng Gao, Ling Cai, Nelli Khudaverdyan, Burak Çetin, Dinshaw J. Patel, Yinsheng Wang, Qiang Cui, Brian D. Strahl, Or Gozani, Kyle M. Miller, Seán E. O’Leary, Paul A. Wade, Gang Greg Wang, Jikui Song

**Affiliations:** 1 Department of Biochemistry, University of California, Riverside, CA USA; 2 Lineberger Comprehensive Cancer Center, University of North Carolina at Chapel Hill School of Medicine, Chapel Hill, NC USA; 3 Department of Biochemistry and Biophysics, University of North Carolina at Chapel Hill School of Medicine, Chapel Hill, NC USA; 4 Division of Intramural Research, Epigenetics and Stem Cell Biology Laboratory, National Institute of Environmental Health Sciences, Research Triangle Park, Durham, NC USA; 5 Department of Molecular Biosciences, LIVESTRONG Cancer Institute of the Dell Medical School, Institute for Cellular and Molecular Biology, University of Texas at Austin, Austin, TX USA; 6 Department of Biology, Stanford University, Stanford, CA USA; 7 Environmental Toxicology Graduate Program, University of California, Riverside, CA USA; 8 Cell, Molecular, and Developmental Biology Graduate Program, University of California, Riverside, Riverside, CA USA; 9 Structural Biology Program, Memorial Sloan Kettering Cancer Center, New York, NY USA; 10 Department of Chemistry, University of California, Riverside, CA USA; 11 Departments of Chemistry, Physics and Biomedical Engineering, Boston University, Boston, MA USA

**Keywords:** DNA methylation, X-ray crystallography

## Abstract

DNA methylation and trimethylated histone H4 Lysine 20 (H4K20me3) constitute two important heterochromatin-enriched marks that frequently cooperate in silencing repetitive elements of the mammalian genome. However, it remains elusive how these two chromatin modifications crosstalk. Here, we report that DNA methyltransferase 1 (DNMT1) specifically ‘recognizes’ H4K20me3 via its first bromo-adjacent-homology domain (DNMT1_BAH1_). Engagement of DNMT1_BAH1_-H4K20me3 ensures heterochromatin targeting of DNMT1 and DNA methylation at LINE-1 retrotransposons, and cooperates with the previously reported readout of histone H3 tail modifications (i.e., H3K9me3 and H3 ubiquitylation) by the RFTS domain to allosterically regulate DNMT1’s activity. Interplay between RFTS and BAH1 domains of DNMT1 profoundly impacts DNA methylation at both global and focal levels and genomic resistance to radiation-induced damage. Together, our study establishes a direct link between H4K20me3 and DNA methylation, providing a mechanism in which multivalent recognition of repressive histone modifications by DNMT1 ensures appropriate DNA methylation patterning and genomic stability.

## Abstract (web summary)

How histone modifications crosstalk with DNA methylation to regulate epigenomic patterning and genome stability in mammals remains elusive. Here, the authors show that DNA methyltransferase DNMT1 is a reader for histone H4K20 trimethylation via its BAH1 domain, which leads to optimal maintenance of DNA methylation at repetitive LINE-1 elements.

## Introduction

The eukaryotic genome is organized into different functional compartments, with the assembly of heterochromatin regulated by both DNA methylation and repressive histone modifications, such as H3 trimethylated at lysine 9 (H3K9me3) and H4 trimethylated at lysine 20 (H4K20me3)^1^. The signaling cascades invoked by these modifications coordinately underpin fundamental biological processes concerning genome compartmentalization, gene silencing, genomic stability, cell differentiation, and development^2,3^. In cancer, depletion of H4K20me3 is closely associated with DNA hypomethylation in repetitive sequences, such as long interspersed nuclear element-1 (LINE-1)^4^, resulting in genomic instability and/or aberrant gene expression^5–7^. Dysregulation of H4K20me3 and DNA methylation has also been associated with neurological and developmental disorders, such as fragile X syndrome^8,9^ and Hutchinson–Gilford Progeria Syndrome (HGPS)^10^. However, it remains far from clear how these two gene-repressive epigenetic modifications cooperate in determining specific chromatin states during normal and pathological development.

**Fig. 1:**
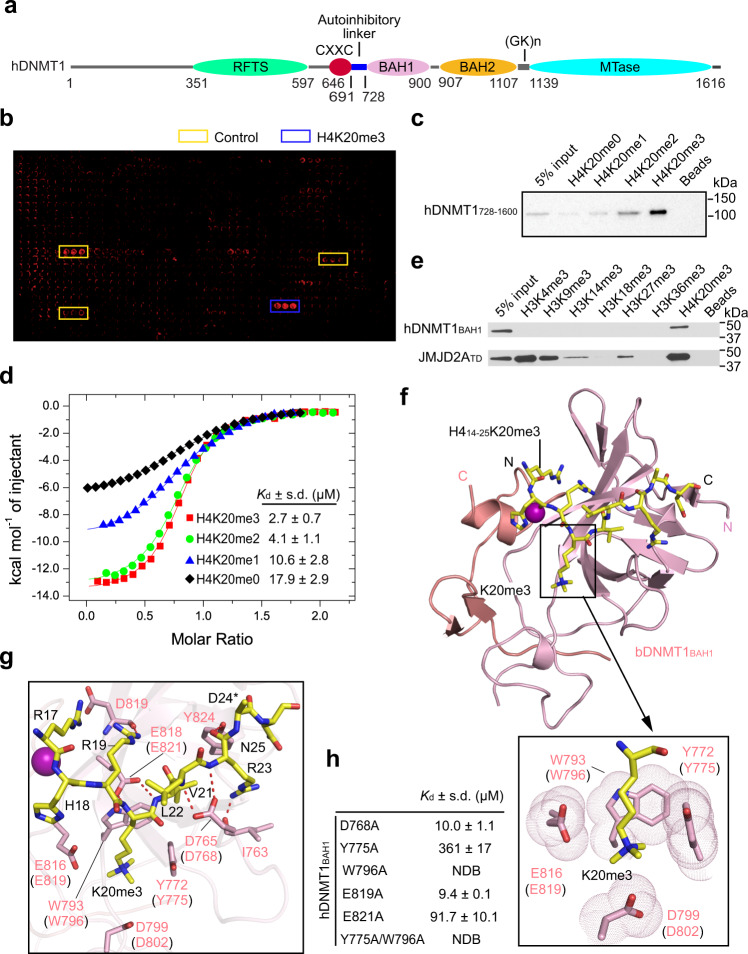
Biochemical and structural analysis of the DNMT1 BAH1–H4K20me3 interaction. **a** Domain architecture of human DNMT1 (hDNMT1), with individual domains delimited by residues numbers. **b** Histone peptide array analysis of the interactions between hDNMT1_728–1600_ and histone peptides. **c** Peptide pull-down analysis of the interactions of hDNMT1_728–1600_ with different H4K20me peptides. The experiment was repeated three times with consistent results. **d** ITC-binding assays for hDNMT1_BAH1_, titrated with different H4K20me peptides. **e** Peptide pull-down analysis of the interactions between hDNMT1_BAH1_ and different tri-methylated histone peptides. The experiment was repeated three times with consistent results. **f** Crystal structure of bDNMT1_BAH1_ (light pink) and H4_14–25_K20me3 peptide (yellow), with the H4K20me3-binding pocket shown in the expanded view (boxed). The last two β-strands, which were domain-swapped during crystallization, are colored in salmon. The zinc ion is shown as a purple sphere. **g** Close-up view of the intermolecular interactions between bDNMT1_BAH1_ and the H4_14–25_K20me3 peptide. The equivalent pocket residues in hDNMT1_BAH1_ are labeled in parentheses in (**f**, **g**). The hydrogen bonds are shown as dashed lines. *The side chain of H4 D24, except for the Cβ atom, is untraceable. **h** *K*_d_ values of hDNMT1_BAH1_ mutants and H4_14–25_K20me3 peptides, derived from the ITC binding assays. NDB, no detectable binding. Source data are provided as a Source Data file.

In mammals, DNA methylation is stably propagated by DNA methyltransferase 1 (DNMT1) during mitotic division^11,12^. DNMT1-mediated DNA methylation maintenance is supported in part by its enzymatic preference for hemimethylated CpG DNA, the so-called maintenance methylation activity^11^. In addition, increasing evidence has suggested a role of de novo methylation activity of DNMT1 in maintaining DNA methylation patterns at H3K9me2/3-enriched^13^ or paternal imprinting control regions^14^. DNMT1 contains a C-terminal methyltransferase (MTase) domain, preceded by several regulatory domains including a replication-foci-targeting sequence (RFTS), a CXXC zinc finger domain and a pair of bromo-adjacent homology (BAH) domains (Fig. 1a)^15–18^. Previous studies have demonstrated that the RFTS and CXXC domains serve to “sense’ specific chromatin cues, which in turn influences DNA methylation maintenance via allosteric regulations^15,17–21^. For instance, the DNMT1 RFTS domain (DNMT1_RFTS_) recognizes ubiquitinated histone H3 (H3Ub)^22–25^ and PCNA-associated factor 15 (PAF15)^26,27^ during S phase, which leads to cell cycle-specific chromatin targeting and enzymatic stimulation of DNMT1^23,26,28^. Our recent study further demonstrated that DNMT1_RFTS_ also directly recognizes H3K9me3, thereby reinforcing the DNMT1_RFTS_-H3Ub readout for the DNA methylation maintenance at heterochromatic regions^29^. Likewise, the DNMT1 CXXC domain specifically recognizes unmodified CpG DNA to control DNMT1-mediated de novo DNA methylation^15,30^. It has been demonstrated that a ~30-amino acid-long linker connecting the CXXC and BAH domains, termed autoinhibitory linker (Fig. 1a)^15^, plays a pivotal role in the transition of DNMT1 between different conformational and functional states^15,18^. However, the mechanism by which these or other regulatory elements are coordinated in fine-tuning the activity of DNMT1 at discrete chromatin regions remains poorly understood.

The BAH domain belongs to an evolutionarily conserved class of reader modules for histone and non-histone proteins^31^. Our previous study identified that the BAH domain of origin recognition complex subunit 1 (ORC1_BAH_) specifically recognizes H4K20 dimethylation (H4K20me2), thereby regulating ORC1-chromatin association and DNA replication initiation^32^. More recently, the BAH domains of two homologous plant proteins, EARLY BOLTING IN SHORT DAYS (EBS) and SHORT LIFE (SHL), effector of polycomb repression 1 (EPR-1) in *Neurospora crassa*, as well as BAH domain and coiled-coil containing 1 (BAHCC1) in human cells, have been shown to mediate Polycomb gene repression via specific readout of H3K27 trimethylation (H3K27me3)^4,33–35^. In DNMT1, the two BAH domains are both associated with the MTase domain to form an integrated structural unit^15^. Although previous studies indicated that these domains play a role in targeting DNMT1 to replication foci^14,36^, their functions remain uncharacterized to date.

To elaborate how the BAH domains regulate DNMT1-mediated DNA methylation, we examined the histone binding activity of DNMT1 BAH domains and its relationship to the chromatin association and enzymatic activity of DNMT1. We identified the first BAH domain of DNMT1 (DNMT1_BAH1_) as a reader for H4K20me3. Strikingly, the H4K20me3 binding by DNMT1_BAH1_ causes displacement of the autoinhibitory linker, which frees DNMT1 from the linker-mediated autoinhibition and leads to allosteric stimulation of DNMT1, reminiscent of what was previously observed for the DNMT1_RFTS_–H3K9me3Ub interaction^23,29^. Consistently, single-molecule Förster resonance energy transfer (smFRET) analysis revealed that the DNMT1_RFTS_–H3K9me3Ub and DNMT1_BAH1_–H4K20me3 interactions both trigger fast conformational dynamics of DNMT1, promoting the transition of DNMT1 into an open conformation. In cells, the DNMT1_BAH1_ mutation, which is defective in recognition of H4K20me3 and yet hyperactive due to the disrupted association between DNMT1_BAH1_ and the autoinhibitory linker, exerts a dual effect on DNA methylation—it causes DNA hypomethylation within the H4K20me3-positive LINE-1, but DNA methylation gains at genomic regions lacking H4K20me3, thereby demonstrating a role for DNMT1_BAH1_ in shaping the landscape of DNA methylation. Finally, we also found that RFTS- and BAH1-mediated DNMT1 regulations cooperate to maintain proper levels of DNA methylation and genome stability of cells. Together, this work establishes a direct link between H4K20me3 and DNMT1-mediated DNA methylation, providing mechanistic insights into DNA methylation maintenance at LINE-1 and other heterochromatic regions.

## Results

### The DNMT1_BAH1_ module is a reader for H4K20me3

To explore the putative reader activity of DNMT1’s BAH modules, we performed histone-binding screening using a C-terminal fragment of hDNMT1, comprised of the BAH and MTase domains (residues 728–1600, hDNMT1_728–1600_; Fig. 1a), on a peptide microarray containing 384 unmodified or uniquely modified histone peptides. We found that DNMT1_728–1600_ binds to the H4K20me3 peptide with the highest affinity (Fig. 1b). This observation was subsequently confirmed by individual peptide pull-down assays, in which GST-tagged hDNMT1_728–1600_ showed a strong preference for H4K20me3 and H4K20me2 over H4K20me1 and H4K20me0 (Fig. 1c). Peptide pull-down and isothermal titration calorimetry (ITC) assays further narrowed down the interaction region to the BAH1 domain (residues 728–900, hDNMT1_BAH1_) (Fig. 1d and Supplementary Fig. 1a and Supplementary Table 1), with preference for H4K20me3 over other lysine-trimethylated histone peptides (Fig. 1e and Supplementary Fig. 1b). Along the line, titration of hDNMT1_BAH1_ with the H4_14–25_K20me3 and H4_14–25_K20me2 peptides gave dissociation constants (*K*_d_) of 2.7 and 4.1 μM, respectively, compared to the *K*_d_ values of 10.6 and 17.9 μM determined for hDNMT1_BAH1_ with H4_14-25_K20me1 and H4_14–25_K20me0, respectively (Fig. 1d and Supplementary Table 1). The binding affinities of hDNMT1_BAH1_ for H4K20me3 and H4K20me2 are comparable with those of previously identified H4K20me3 and H4K20me2 readers, notably the JMJD2A tudor domain (JMJD2A_TD_)^37^ and the ORC1_BAH_ domain^32^, respectively (Supplementary Fig. 1c, d and Supplementary Table 1). Together, these data establish that the hDNMT1_BAH1_ domain recognizes highly methylated H4K20 with a preference for H4K20me3.

### Structural details for the Interaction between DNMT1 BAH1 Domain and H4K20me3

To understand the structural basis for the DNMT1_BAH1_–H4K20me3 interaction, we determined the crystal structure of the BAH1 domain of bovine DNMT1 (bDNMT1_BAH1_), which shares ~94% sequence identity with hDNMT1_BAH1_ (Supplementary Fig. 1e), in complex with the H4_14–25_K20me3 peptide at 2.65 Å resolution (Fig. 1f and Table 1). The structure of bDNMT1_BAH1_ reveals a twisted β-barrel resembling hDNMT1_BAH1_ (Fig. 1f and Supplementary Fig. 1f, g). The H4_14–25_K20me3 peptide extends along one end of the β-barrel (Fig. 1f), with the side chain of H4K20me3 inserted into a pocket formed by Y772, W793, D799, and E816 of bDNMT1_BAH1_ (Fig. 1f, boxed). Flanking the H4_14–25_K20me3 peptide, the side chains of bDNMT1_BAH1_ D765 and E818 each form a bidentate hydrogen bond with the backbone amides of H4, and the backbone of bDNMT1_BAH1_ D765 is further hydrogen bonded to the side chain of H4 R23 (Fig. 1g). Additional intermolecular interactions involve bDNMT1_BAH1_ I763, D819, and Y824 and H4 R17, R19, and R23, which mediate hydrogen bonding or electrostatic attractions (Fig. 1g).

**Table 1:** Data collection and refinement statistics.

	bDNMT1_BAH1_–H4K20me2 (PDB: 7LMM)	bDNMT1_BAH1_–H4K20me3 (PDB: 7LMK)
*Data collection*		
Space group	P 2 2_1_ 2_1_	P 2 2_1_ 2_1_
*Cell dimensions*		
*a*, *b*, *c* (Å)	70.7, 80.3, 130.4	71.1, 81.4, 129.6
*α*, *β*, *γ* (°)	90.00, 90.00, 90.00	90.00, 90.00, 90.00
Resolution (Å)	34.91–2.80(2.90–2.80)^a^	81.37–2.65 (2.71–2.65)
*R*_merge_	0.117 (0.968)	0.073 (1.632)
*I/σ*(*I*)	20.8 (2.26)	15.7 (1.0)
*CC*_1/2_ Completeness (%)	0.998 (0.866) 99.5 (99.8)	0.998 (0.404) 98.8 (97.6)
Redundancy	12.9 (13.2)	4.4 (4.4)
*Refinement*		
No. of reflections	18,807	22,119
*R*_work_/*R*_free_	0.207/0.267	0.206/0.258
*No. of atoms*		
Protein	5168	5114
Zn^2+^	4	4
Water	36	29
*B factors (Å*^*2*^*)*		
Protein	68.4	83.1
Zn^2+^	43.0	68.8
Water	45.4	66.8
*R.M.S. deviations*		
Bond lengths (Å)	0.005	0.004
Bond angles (°)	0.713	0.691

^a^Values in parentheses are for highest-resolution shell. Each structure was determined using the dataset collected from a single crystal.

To elucidate the H4K20me3-binding specificity, we also determined the crystal structure of bDNMT1_BAH1_ in complex with the H4_14–25_K20me2 peptide at 2.8 Å resolution (Supplementary Fig. 2a and Table 1). The structure of bDNMT1_BAH1_–H4K20me2 reveals a similar interaction mode as that of bDNMT1_BAH1_–H4K20me3, with the side chain of H4K20me2 surrounded by the same set of H4K20me3-binding residues (Supplementary Fig. 2a, boxed). Distinct from the previously determined structure of the ORC1_BAH_–H4K20me2 complex in which the dimethylammonium group of H4K20me2 forms a hydrogen bond with a surrounding glutamate (ORC1_BAH_ E93) (Supplementary Fig. 2b)^32^, the side chain of H4K20me2 in the bDNMT1_BAH1_ complex is not within a hydrogen-bond distance with the side-chain carboxylates of D799 and E816 (Supplementary Fig. 2a), thus explaining why hDNMT1_BAH1_ shows a relative lower binding affinity for H4K20me2 than for H4K20me3.

Sequence alignment of the DNMT1 BAH1 domain across different species revealed that the H4K20me2/3-binding sites fall into the most conserved regions (Supplementary Fig. 1e). Mutation of hDNMT1_BAH1_ at or near the corresponding H4K20me3-binding pocket residues (D768A, Y775A, E819A, and E821A; Supplementary Fig. 1e) significantly reduced its binding affinity for the H4_14–25_K20me3 or H4_14–25_K20me2 peptide, while introduction of a W796A mutation largely abolishes the hDNMT1_BAH1_–H4_14–25_K20me3 or hDNMT1_BAH1_–H4_14–25_K20me2 binding (Fig. 1h and Supplementary Fig. 2c–l and Supplementary Table 1). These observations suggest that DNMT1 has evolved with a conserved mechanism for H4K20me2/3 recognition.

### H4K20me3 binding displaces the autoinhibitory linker for enzymatic stimulation

**Fig. 2:**
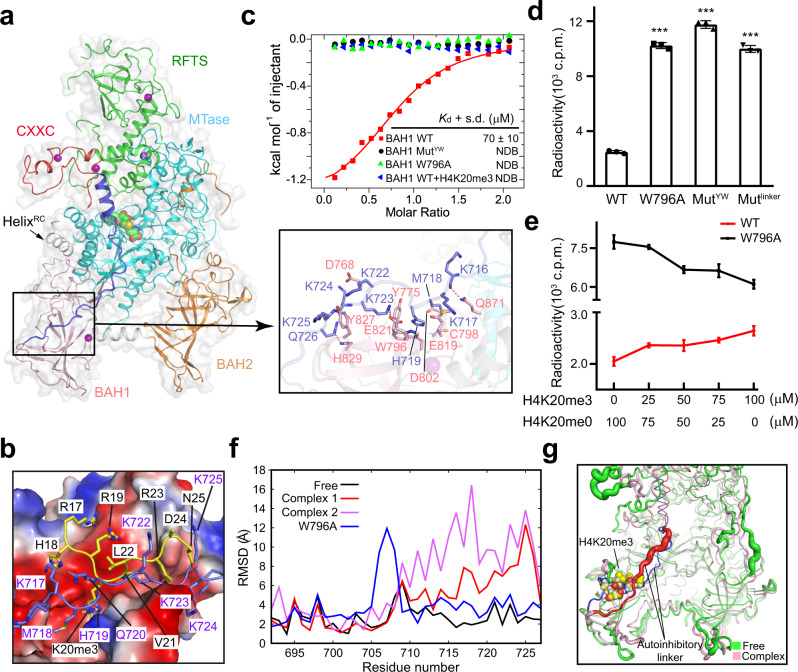
H4K20me3 binding allosterically stimulates DNMT1 activity. **a** Ribbon and surface diagram of the structure of hDNMT1_351–1600_ (PDB 4WXX [10.2210/pdb4WXX/pdb]), with individual segments color coded. The RFTS–CXXC linker helix is denoted as Helix^RC^. The detailed interactions between the autoinhibitory linker and the BAH1 domain are shown in the expanded view. The hydrogen bonds are shown as dashed lines. **b** Close-up view of the structural overlay between hDNMT1_351–1600_ (PDB 4WXX [10.2210/pdb4WXX/pdb]) and the bDNMT1_BAH1_–H4K20me3 complex, with the autoinhibitory linker and the H4K20me3 peptide colored in slate and yellow, respectively, and the corresponding binding site in hDNMT1_351–1600_ shown as electrostatic surface. The bDNMT1_BAH1_ domain was removed for clarity. **c** ITC binding analysis of the interaction between hDNMT1_BAH1_, either WT, W796A, or Mut^YW^, and the autoinhibitory linker peptide, in the absence of presence of the H4_14–25_K20me3 peptide. **d** DNA methylation activity of hDNMT1_351-1600_, WT, W796A, Mut^YW^, or Mut^linker^. Data are mean ± s.d. (*n* = 3 biological replicates). Statistical analysis for WT vs. mutants used two-tailed Student’s *t* test. ***p < 0.001. **e** H4K20me3-dependent DNA methylation activity of hDNTM1_351–1600_, either WT or W796A. The total concentration of H4_14–25_K20me0 and H4_14–25_K20me3 peptides were maintained at 100 μM to eliminate the substrate-competing effects of the H4 peptides. Data are mean ± s.d. (*n* = 3 biological replicates). **f** The RMSD values obtained for the autoinhibitory linker in the structural models of WT (PDB 4WXX [10.2210/pdb4WXX/pdb]) or W796A hDNMT1_351–1600_ and the hDNMT1_351–1600_-H4K20me3 complex (complex 1 and complex 2) during 100 ns MD simulations. **g** Sausage view of the RMSF values of the autoinhibitory linker in peptide-free and H4K20me3-bound hDNMT1_351–1600_ (complex 2), colored in blue and red, respectively. Source data are provided as a Source Data file.

Previously, the autoinhibitory linker (residues 692–727; blue in Fig. 2a) was shown to reinforce the RFTS-mediated autoinhibition through both stabilization of the RFTS-MTase association and occlusion of the DNA substrate (Fig. 2a and Supplementary Fig. 3a)^17,18,21^. Interestingly, structural comparison of the bDNMT1_BAH1_–H4K20me3 complex with the histone-free hDNMT1_351–1600_ (PDB 4WXX)^18^ revealed that the H4K20me3-binding site of DNMT1_BAH1_ is shielded by the C-terminal half of the autoinhibitory linker in free hDNMT1_351–1600_, via extensive intramolecular interactions (Fig. 2a, b), implying that the H4K20me3 binding to BAH1 would lead to displacement of the autoinhibitory linker, thereby providing a potential mechanism for releasing DNMT1 from autoinhibition. To explore this possibility, we first tested whether the autoinhibitory linker and H4K20me3 compete against each other for BAH1 binding. Using ITC assays, we found that the hDNMT1_BAH1_ domain binds to a peptide derived from the autoinhibitory linker with a *K*_d_ of ~70 µM (Fig. 2c); this binding was abolished in the presence of a twofold excess of H4K20me3 peptide (Fig. 2c). The ITC assay also reveals that, unlike the hDNMT1_BAH1_ domain alone that binds to H4K20me3 peptide with a *K*_d_ of 2.7 µM (Fig. 1d), the linker-containing DNMT1_351–1600_ only weakly binds to the H4K20me3 peptide, with a *K*_d_ of over 300 µM (Supplementary Fig. 3b). These data establish that the competition for BAH1 binding exists between the H4K20me3 peptide and the autoinhibitory linker in a histone-free form of DNMT1.

To elucidate the functional consequence of H4K20me3 binding, we generated mutants of the BAH1 domain, W796A, and Y775A/W796A (hereafter referred to as Mut^YW^), in which the associations of DNMT1_BAH1_ with both H4K20me3 and the linker peptides are completely abolished (Fig. 2c and Supplementary Fig. 3b). In addition, we generated a sextuple mutation in the autoinhibitory linker, in which the BAH-contacting residues M718, H719, K723, K724, K725, and Q726 were each replaced by alanine (hereafter referred to as Mut^linker^), to reduce the association of the autoinhibitory linker with the DNMT1 BAH1 domain without disruption of the H4K20me3-binding affinity of the latter. ITC analysis revealed that the Mut^linker^ mutation enhanced the hDNMT1_351–1600_–H4K20me3 binding affinity by two to threefold (Supplementary Fig. 3b and Supplementary Table 1), consistent with the notion that the autoinhibitory linker and H4K20me3 compete for BAH1 binding. Furthermore, in vitro DNA methylation assays indicate that W796A, Mut^YW^, and Mut^linker^ mutations all lead to enhanced enzymatic activity of DNMT1 by four to fivefold (Fig. 2d), suggesting that disruption of the interactions between the BAH1 domain and the autoinhibitory linker helps relieve DNMT1’s autoinhibition. Consistently, under fixed peptide concentration, incubation with an increasing ratio of H4_14–25_K20me3 vs. H4_14–25_K20me0 gradually increased the DNA methylation efficiency of wild-type (WT) hDNMT1_351–1600_, but not the W796A mutant (Fig. 2e). The caveat of these studies is that the histone peptides used for the assays may not recapitulate the multivalent DNMT1–nucleosome interaction within the chromatin environment.

To further illustrate the H4K20me3 binding-triggered conformational change, we performed molecular dynamics (MD) simulation studies on hDNMT1_351–1600_, either WT or W796A, and the structural model of the hDNMT1_351–1600_–H4K20me3 complex. The hDNMT1_351–1600_–H4K20me3 complex, simulated under two independent trajectories, gave substantially elevated root-mean-square deviation (RMSD) and root-mean-square fluctuation (RMSF) for the autoinhibitory linker than free-state hDNMT1_351–1600_ (Fig. 2f, g and Supplementary Fig. 3c). Likewise, a notable increase in the RMSD and RMSF of the autoinhibitory linker was observed for the W796A mutant (Fig. 2f and Supplementary Fig. 3c), further supporting the notion that linker detachment leads to the enzymatic activation of DNMT1.

### BAH1–H4K20me3 and RFTS–H3K9me3Ub2 bindings both drive to a conformationally dynamic state of DNMT1

**Fig. 3:**
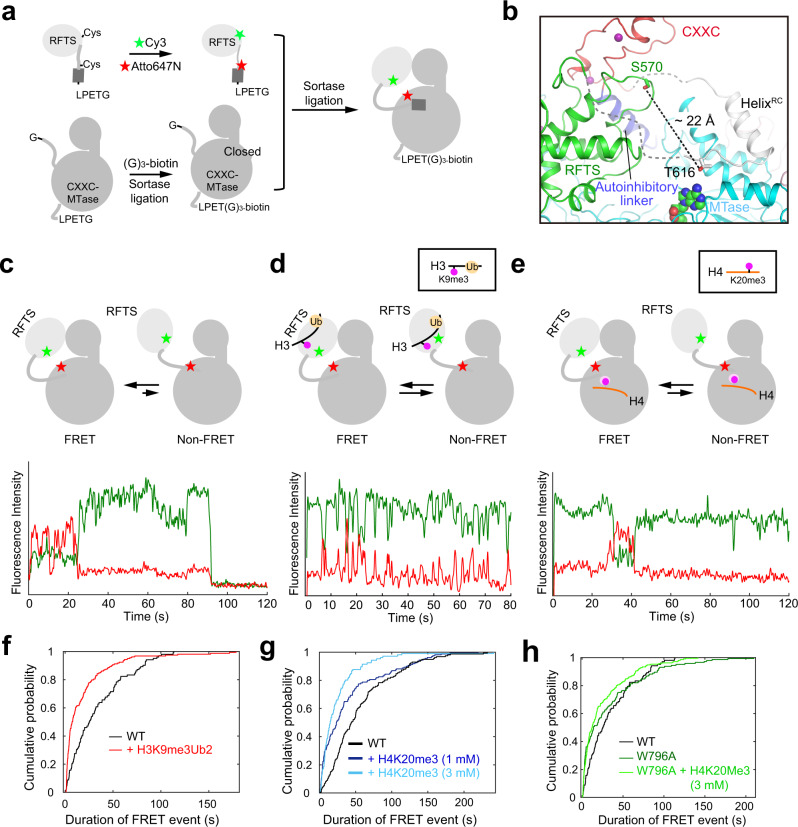
smFRET analysis of the conformational dynamics of DNMT1 upon H3K9me3Ub2 or H4K20me3 binding. **a** Schematic view of the preparation of Cy3/Atto647N-labeled hDNMT1 fragment (hDNMT1^CY^) for smFRET analysis. The Cy3 and Atto647N dyes are shown as green and red stars. The Helix^RC^ is shown as cylinder in dark gray. The RFTS domain and the rest of DNMT1 are colored light gray and gray, respectively. **b** Close-up view of the autoinhibitory state of hDNMT1_351–1600_ (PDB 4WXX [10.2210/pdb4WXX/pdb]), with the distance between S570 and T616 indicated by a dashed line in black. The linker connecting the RFTS and CXXC domains is in gray, with dashed lines indicating disordered regions. **c** (Top) Model for the conformational transition between autoinhibited and activated hDNMT1^CY^. (Bottom) Representative smFRET trace observed for peptide-free wild-type hDNMT1^CY^. **d** (Top) Model for the conformational transition between autoinhibited and activated conformations of H3K9me3Ub2-bound hDNMT1^CY^. The H3K9me3 and ubiquitin marks are shown as magenta and wheat spheres, respectively. (Bottom) Representative smFRET trace observed for hDNMT1^CY^ in the presence of H3K9me3Ub2. **e** (Top) Model for the conformational transition between autoinhibited and activated conformations of H4K20me3-bound hDNMT1^CY^. The H4K20me3 mark is shown as magenta sphere. (Bottom) Representative smFRET trace observed for hDNMT1^CY^ in the presence of H4K20me3. **f** Cumulative probability of the durations of FRET events observed for hDNMT1^CY^, in the absence (black) or presence (red) of H3K9me3Ub2 peptide (97 and 93 molecules, respectively). **g** Cumulative probability of the durations of FRET events observed for hDNMT1^CY^, in the absence (black, 150 molecules) or presence of 1 mM (blue, 119 molecules), or 3 mM (cyan, 88 molecules) H4K20me3 peptide. **h** Cumulative probability of the durations of FRET events observed for peptide-free WT hDNMT1^CY^ (black, 97 molecules), compared with W796A hDNMT1^CY^ in the absence (dark green, 95 molecules) or presence (light green, 164 molecules) of 3 mM H4K20me3 peptide.

The BAH1–H4K20me3 binding-mediated allosteric activation of DNMT1 is reminiscent of the previously reported RFTS–H3K9me3Ub2 readout^29^. We therefore asked how the BAH1–H4K20me3 and RFTS–H3K9me3Ub2 interactions crosstalk. Toward this direction, we performed smFRET experiments to interrogate how the RFTS–H3K9me3Ub and BAH1–H4K20me3 interactions impact the conformation of DNMT1. To measure the inter-domain movement between the RFTS and MTase domains, we introduced two mutation sites, one on the RFTS domain (S570C) and the other on the C-terminal end of the RFTS–CXXC linker (T616C), to a hDNMT1 fragment (residues 351–639 followed by a LPETG sequence) for statistical labeling with an equimolar mixture of FRET donor (Cy3) and acceptor (Atto647N) (Fig. 3a). Such a labeling strategy permitted close proximity between the two fluorophores in histone-free hDNMT1, taking advantage of the fact that the RFTS–CXXC linker is C-terminally anchored to the MTase domain through helical packing (Fig. 3b). This hDNMT1 fragment was then sortase-ligated^38^ with a C-terminally biotinylated hDNMT1 fragment, creating a Cy3,Atto647N-labeled hDNMT1 fragment encompassing residues 351–1606 (denoted hDNMT1^CY^) for smFRET observation (Fig. 3c–e).

SmFRET between the two fluorophore labels was observed for wild-type hDNMT1^CY^, typically characterized by stable FRET observed from the beginning of the movie until acceptor photobleaching (Fig. 3c), which is consistent with the closed, autoinhibitory structure of the enzyme. Addition of the H3K9me3Ub2 peptide to surface-immobilized hDNMT1^CY^ led to a dramatic reduction in the duration of FRET events, with a population of molecules cycling between non-FRET and FRET states (Fig. 3d). In comparison with the peptide-free FRET events, which had a single-exponential distribution of event durations, addition of the peptide therefore led to appearance of an additional, faster, exponential phase (Fig. 3f). These data confirm a previous MD analysis that showed enhanced mobility of the RFTS domain upon the H3Ub2 binding^23^, and suggest that the H3K9me3Ub2 binding promotes DNMT1 transition from a stable, autoinhibitory state to an activated state permitting fast “closed”–“open” conformational interconversion. Intriguingly, the presence of increasing concentrations of H4K20me3 peptide, but not H3K4me3 peptide, leads to a similar reduction in the duration of FRET events (Fig. 3e, g and Supplementary Fig. 3d–f), supporting the model that H4K20me3 binding influences the conformational dynamics of DNMT1. The W796A mutant showed an intrinsically reduced duration of FRET events (Fig. 3h and Supplementary Fig. 3g–i), regardless of the addition of H4K20me3 peptide (Fig. 3h), which reinforces the notion that the hyperactivity of this mutant arises from the detachment of the autoinhibitory linker from the BAH1 domain of DNMT1. Together, these smFRET data support a model where specific interactions, modulated by peptide binding to the RFTS domain or the BAH1 domain, gate access to a conformationally dynamic, activated state of DNMT1.

### BAH1–H4K20me3 binding potentiates CpG methylation deposition at H4K20me3-demarcated genomic regions, with the most striking effect seen at LINE-1 elements

**Fig. 4:**
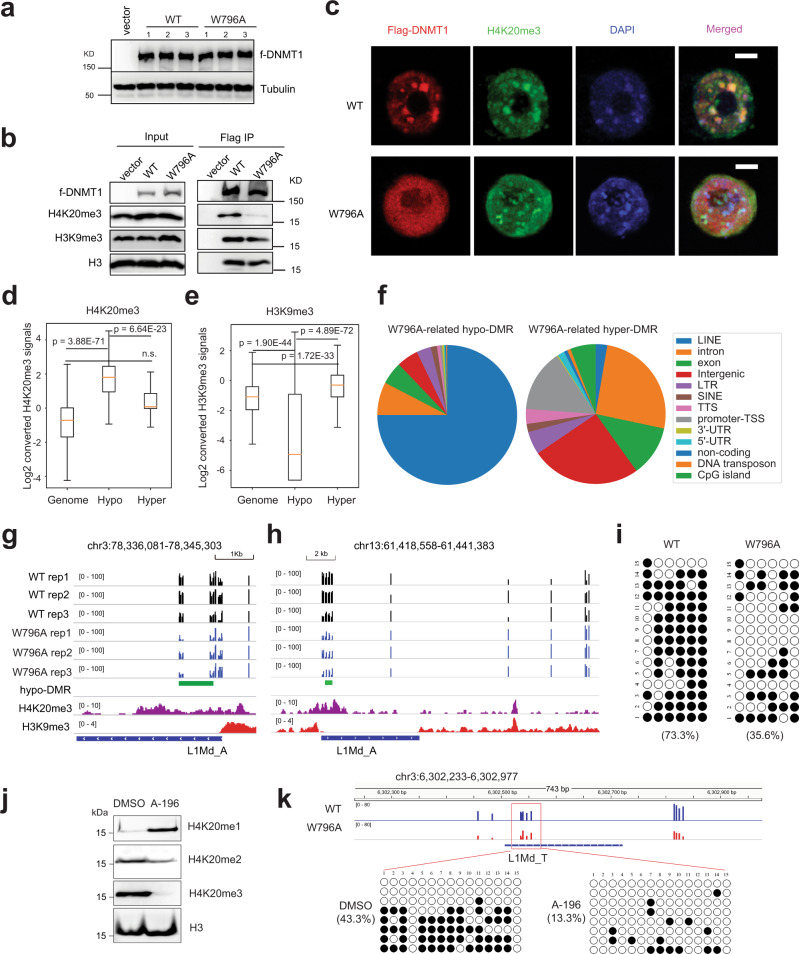
The BAH1–H4K20me3 binding regulates DNMT1-mediated methylation at the H4K20me3-enriched genomic regions. **a** Immunoblots of the indicated Flag-tagged DNMT1 after its stable reconstitution into 1KO-ESCs among the independently derived cell lines. The experiment was repeated three times with consistent results. **b** CoIP (right panels) detecting association of the indicated Flag-tagged DNMT1 with H4K20me3 or histone H3. Left panels are immunoblots of input. The experiment was repeated three times with consistent results. **c** Representative confocal immunofluorescence images revealing localization of the indicated DNMT1 (Flag-tagged, red), H4K20me3-dense loci (green), and chromatin (stained by DAPI, blue) in the 1KO-ESC stable expression lines, synchronized in S phase. Scale bar, 5 µm. The experiment was repeated three times with consistent results. **d**, **e** Box plots showing the levels of H4K20me3 (**d**) or H3K9me3 (**e**) in ESCs, genome-wide (left) or within DMRs found associated with the W796A mutation of DNMT1 relative to WT, either hypo (middle) or hyper-methylated (right) (for panels **d**, **e**, *n* = 422 for Hypo, 377 for Hyper and 422 for randomized genome regions as a control). DMR calling is performed using DSS. Box-and whisker plots depict 25–75% in the box, whiskers are 10–90%, and median is indicated. Statistical analysis used two-tailed Student’s *t* test. **f** Pie chart showing distribution of the DMRs associated with the W796A-mutated DNMT1 relative to WT, either hypo (left) or hyper-methylated (right), among the indicated genomic feature. DMR calling is performed using DSS. **g**, **h** Representative IGV views show CpG methylation levels at the called W796A-asociated hypo-DMRs (green bar), either located in the chromosome 3 (**g**) or 13 (**h**), among three replicated 1KO-ESC lines with stable expression of WT (black) or the BAH1-mutated (blue) DNMT1. Cytosines covered by at least five reads according to eRRBS data are shown, with each site designated by a vertical line. The bottom panels show IGV view of H4K20me3 (purple) and H3K9me3 (red) in WT ESCs, and position of LINE-1. **i** Bisulfite sequencing analysis of CpG methylation at the regulatory region of L1 in cells transfected with DNMT1^W796A^ or DNMT1^WT^. **j** Immunoblotting of H4K20 methylation (H4K20me1/2/3; with H3 as control) in E14 ESCs treated with 10 µM A-196, vs. DMSO, for three days. The experiment was repeated twice with consistent results. **k** Site-targeted bisulfite sequencing results (bottom) revealed hypomethylation of a LINE-1 element (L1Md_T) in E14 ESCs treated with A-196 vs. DMSO. Top panel shows an IGV view of eRRBS data at this L1Md_T site, with a red box marking the CpG-containing region examined by site-targeted bisulfite sequencing. Source data are provided as a Source Data file.

Given that *Dnmt1* deletion is lethal to dividing somatic cells, but not mouse ES cells^39^, we turned to mouse ES cells to examine the role of DNMT1_BAH1_ in regulating DNA methylation maintenance. Using our recently reported gene complementation system^29^, we introduced comparable levels of exogenous DNMT1^WT^ or a BAH1-defective mutant (DNMT1^W796A^) into Dnmt1-knockout mouse embryonic stem cells (1KO-ESCs) (Fig. 4a). In agreement with the above structural and biophysical assays of DNMT1_BAH1_, we found that DNMT1^WT^ significantly co-precipitated with H4K20me3 (Fig. 4b), and as previously observed^25^, co-localizes with punctate DAPI-dense heterochromatin foci marked with H4K20me3 (Fig. 4c and Supplementary Fig. 4; upper panels). In contrast, DNMT1^W796A^ shows much reduced co-precipitation with H4K20me3 (Fig. 4b) and the reduced H4K20me3 co-staining (Fig. 4c and Supplementary Fig. 4; lower panels).

Further, we carried out genome-wide methylation profiling with enhanced reduced representation bisulfite sequencing (eRRBS), which showed nearly complete bisulfite conversion (99.79–99.83%; Supplementary Fig. 5a), with at least 5-fold coverage for ~5–8 million of CpG sites in all samples (Supplementary Data 1). First, overall DNA methylation levels were found similar among independently derived 1KO-ESC lines with the reconstituted DNMT1^WT^ or DNMT1^W796A^ (Supplementary Fig. 5b, c and Supplementary Data 1), which was verified by global cytosine methylation quantification by liquid chromatography–mass spectrometry (LC–MS) (Supplementary Fig. 5d). Close inspection of eRRBS data, however, revealed that there was a slight but significant decrease in overall CpG methylation at the H4K20me3-demarcated genomic regions in 1KO-ESCs expressing DNMT1^W796A^, relative to the DNMT1^WT^ controls, with the observed decreases being more apparent and significant at regions with the highest H4K20me3 (Supplementary Fig. 5e). In contrast, there was a slight but significant increase of overall CpG methylation at randomized control regions that lack H4K20me3 (Supplementary Fig. 5f, g). We have further defined differentially methylated regions (DMRs), either hypo-methylated (hypo-DMR) or hyper-methylated (hyper-DMR), by comparing eRRBS profiles of 1KO-ESCs with DNMT1^W796A^ vs. DNMT1^WT^. With two independent DMR calling methods (Supplementary Data 2 and 3), we consistently observed a significant enrichment of the DNMT1^W796A^-associated hypo-DMRs at H4K20me3-marked regions, relative to genome background or DNMT1^W796A^-associated hyper-DMRs (Fig. 4d and Supplementary Fig. 5h). Meanwhile, compared to genome background, the DNMT1^W796A^-associated hypo-DMRs were found significantly depleted from the H3K9me3-marked regions (Fig. 4e and Supplementary Fig. 5i), indicating that different stimulating mechanisms exist to ensure optimal DNA methylation among H3K9me3- and H4K20me3-demarcated heterochromatin. As a control, CUT&RUN of H4K20me3 demonstrated no change of overall H4K20me3 levels in these cells, as exemplified by retrotransposon elements known to be targeted by H4K20me3^40,41^ (Supplementary Fig. 5j, k). These genomic profiling results are in agreement with a notion revealed by our in vitro studies that the W796A mutation of BAH1 has dual effects, with one causing the impaired CpG methylations within H4K20me3-marked regions (due to disrupted association of DNMT1 with H4K20me3) and the other leading to a generally enhanced methylation (due to the reduced association between the mutated BAH1 and the autoinhibitory linker of DNMT1 and hence, hyperactivity of this mutant).

Interestingly, a majority of the DNMT1^W796A^-related hypo-DMRs were found localized within the LINE-1 class of retrotransposon elements (also known as L1; Fig. 4f and Supplementary Fig. 6a), especially the 5′ *cis*-regulatory region of a newly evolved, non-truncated L1MdA subfamily (Fig. 4g, h and Supplementary Fig. 6b and Supplementary Data 4). LINE elements are known to be targeted by H4K20me3 and DNA methylation^40,41^, and in our eRRBS data, had comparable coverage and sequencing depth relative to the Alu/SINE family of repetitive elements and gene-coding regions (Supplementary Fig 6a, right panels). By individual bisulfite sequencing, we verified decreased CpG methylation at the examined regulatory region of L1 in cells with DNMT1^W796A^ relative to DNMT1^WT^ (Fig. 4i). Moreover, we used CRISPR/cas9-based gene editing technology to introduce into E14 ESCs a point mutation of W799A to endogenous Dnmt1 (equivalent to hDNMT1 W796A used above; Supplementary Fig. 6c, d), and subsequent individual bisulfite sequencing also detected decrease in CpG methylation at the examined L1 element (Supplementary Fig. 6e, f). Next, to further test a requirement of H4K20me3 for CpG methylation of LINE-1, we turned to A-196, a selective inhibitor of H4K20 methyltransferases SUV420H1 and SUV420H2^42^. As expected, A-196 treatment of E14 ESCs led to a global loss of H4K20me3, a significant decrease of H4K20me2 and a concomitant increase in H4K20me1 (Fig. 4j), which was concurrent with CpG hypomethylation of the examined L1 as well (Fig. 4k). Altogether, these results support that DNMT1_BAH1_ binding to H4K20me3 potentiates DNMT1-mediated CpG methylation of H4K20me3-demarcated regions, notably those newly evolved non-truncated LINE-1 elements.

### BAH1 and RFTS regulations cooperate in fine-tuning DNMT1 activity

**Fig. 5:**
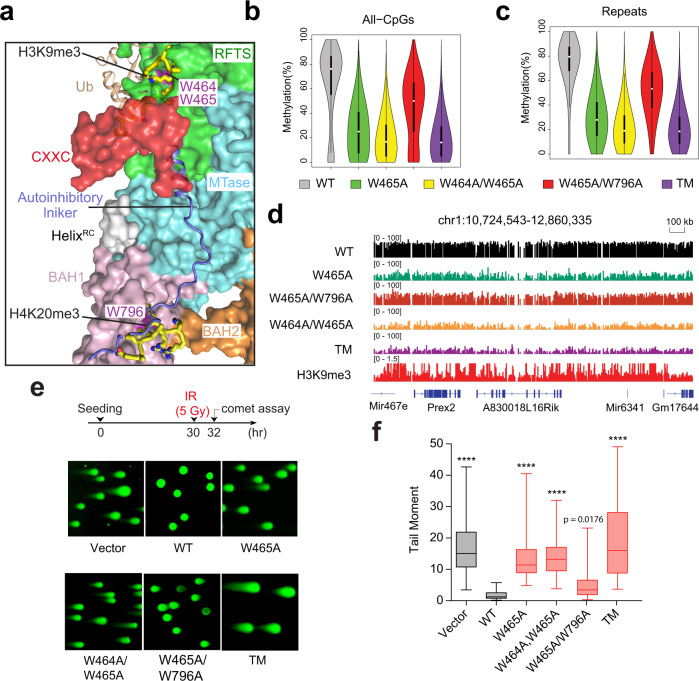
RFTS and BAH1 domains crosstalk in DNMT1-mediated DNA methylation. **a** Close-up view of the structurally overlaid hDNMT1_351–1600_ (PDB 4WXX [10.2210/pdb4WXX/pdb]), bDNMT1_RFTS_ bound to H3K9me3 and ubiquitin (PDB 6PZV [10.2210/pdb6PZV/pdb]), and the bDNMT1_BAH1_–H4K20me3 complexes, with the surface of individual DNMT1 domains color coded in the same fashion as in Fig. 2a. hDNMT1 W464, W465, and W796 are colored purple. The autoinhibitory linker colored blue. The H3K9me3 and H4K20me3 peptides are shown as yellow sticks. The ubiquitin molecule is shown in ribbon representation and colored in wheat. The bDNMT1_RFTS_ and bDNMT1_BAH1_ molecules were removed for clarity. **b**, **c** Violin plots showing distribution of absolute methylation levels for CpG sites with >5 coverage at all CpG sites (**b**) or those within the repeated genomic sequences (**c**) among the indicated cells samples. (*n* = 5,040,986 CpG sites in panel (**b**); *n* = 812,251 repeats in panel (**c**)). White dots are the median and box lines are the first and third quartile of the data. **d** Representative IGV view of CpG methylation at an H3K9me3-marked genomic region located in the chromosome 1 among three replicated 1KO-ESC lines with stable expression of the indicated DNMT1. Cytosines covered by at least ten reads according to eRRBS data (with three replications merged for each group) are shown, with each site designated by a vertical line. **e** Neutral comet assays, experimental scheme (top) and DNA breaks (bottom). **f** Quantified DNA breaks after ionizing radiation (IR) treatment of 1KO-ESC cells reconstituted with vector control or the indicated DNMT1. Box-and-whisker plots in panel depict 25–75% in the box, whiskers are 10–90%, and median is indicated. Data are mean ± s.d. from >100 cells (*n* = 3 biologically independent replicates). One-way ANOVA with post Tukey analysis was used. ****p < 0.0001. The data for vector, WT, W465A, and W464A/W465A were adopted from those published previously^29^.

Our recent study has identified that W464 and W465 within the DNMT1_RFTS_ module are essential for H3K9me3Ub2 recognition (Fig. 5a); introduction of the W464A and W464/W465A mutation leads to severely impaired RFTS–H3K9me3Ub2 binding, and consequently, a large reduction of DNA methylation in cells^29^. To further examine the interplay between the RFTS- and BAH1-mediated regulations of DNMT1, we combined the W796A mutation of DNMT1_BAH1_ together with the RFTS-defective mutation, either W465A or W464A/W465A, and subsequently used such RFTS/BAH1 compound mutants for DNMT1 reconstitution in 1KO-ESCs (Supplementary Fig. 7a). Intriguingly, both LC–MS analysis of cytosine methylation (Supplementary Fig. 7b) and eRRBS (Fig. 5b, c and Supplementary Fig. 7c–e) revealed that, relative to the single mutant of RFTS (DNMT1^W465A^)^29^, the compound mutant of RFTS/BAH1 (DNMT1^W465A/W796A^) displayed a markedly enhanced methylation activity in cells, leading to partial but significant rescue of the global CpG hypomethylation phenotype caused by DNMT1^W465A^, including the RFTS-related defects seen at the H3K9me3-marked regions as exemplified by sub-telomeric regions located at the chromosome 1 (Fig. 5d). Given the intrinsic link between the hyperactivity of DNMT1^W796A^ and the BAH1-mediated DNMT1 activation, these data therefore show that the BAH1-mediated DNMT1 activation may partially rescue the CpG methylation defects caused by the RFTS dysfunction. Note that such a rescue effect was not observed for the RFTS W464A/W465A mutation in the resultant triple mutation of DNMT1^W464A/W465A/W796A^ (referred to as “TM”; Fig. 5b–d and Supplementary Fig. 7b–d), presumably due to the more severe impairment of chromatin targeting capability associated with this mutant^29^.

### RFTS and BAH1 coordinately regulate DNMT1-mediated genomic stabilization

We further challenged 1KO-ESC cells, reconstituted with WT or mutant DNMT1, with ionizing radiation (IR) followed by the neutral comet assay (Fig. 5e). First, compared to 1KO-ESC cells rescued with WT DNMT1, those with vector control or a catalytically inactive DNMT1 mutant (C1226S) showed similar hyper-sensitivity to IR (Supplementary Fig. 7f–h), thus confirming a direct link between DNMT1-mediated DNA methylation and genomic stability. We next examined whether the BAH1 and RFTS mutations crosstalk during the IR response. As previously demonstrated^29^, cells with the RFTS W464A/W465A and W465A mutants displayed severe impairment of IR resistance (Fig. 5e, f). The compound mutation of DNMT1^W464A/W465A/W796A^ (Fig. 5e, f) led to a stronger phenotype of DSB persistence following IR treatment, in line with severe hypomethylation seen with this TM mutation (Fig. 5b–d). In contrast, while cells with the RFTS single mutant (DNMT1^W465A^) exhibited the impaired genome integrity post-treatment of IR, introduction of an additional BAH1 mutation (DNMT1^W465A/W796A^) significantly rescued this defect (Fig. 5e, f), in line with the rescuing effect of the BAH1 W796A mutation manifested in LC–MS- and eRRBS-based methylome analysis (Fig. 5b–d and Supplementary Fig. 7b). Consistently, cell survival analysis revealed that the DNMT1^W465A/W796A^ mutation led to a significant reduction in IR sensitivity in comparison with the DNMT1^W465A^ mutation (Supplementary Fig. 7i). Thus, it is evident that DNMT1-mediated DNA methylation is involved in maintenance of genomic stability, the molecular detail of which awaits further investigation.

## Discussion

The spatio-temporal regulation of DNMT1-mediated DNA methylation is essential for the faithful inheritance of DNA methylation patterns during mitotic division. DNA methylation, in cooperation with other gene silencing mechanisms, such as histone modifications, provides a mechanism for the long-term stability of the heterochromatic state. However, how DNA methylation crosstalks with histone modifications remains elusive. Through a set of structural, biochemical, computational, and cellular analyses, this study addresses two critical questions regarding the functional regulation of DNMT1.

First, this study links the repressive histone modification H4K20me3 directly to the reader activity of DNMT1. Here, we show that DNMT1 directly recognizes H4K20me3 through the regulatory domain BAH1, which in turn mediates the chromatin association and enzymatic activation of DNMT1. The BAH1–H4K20me3 interaction clarifies a putative role of the BAH domains of DNMT1 in genomic targeting^14,36^, and provides a crucial safeguard mechanism for efficient DNA methylation at H4K20me3-enriched regions, in particular the repetitive element LINE-1, thereby providing further insights into the mechanism underlying DNA methylation maintenance and heterochromatin formation. Distinct from the typical Kme3 readout that depends on aromatic or other hydrophobic residues only^43^, the BAH1 domain recognizes H4K20me3 via a pocket formed by mixed aromatic and acidic residues. In the DNA-free state of DNMT1, the H4K20me3-binding site is occluded by the C-terminal portion of the autoinhibitory linker, which likely reinforces the intramolecular RFTS-linker-MTase association (Supplementary Fig. 8a), resulting in a direct link between the H4K20me3 binding and enzymatic activation of DNMT1. It is worth noting that de novo P767T mutation of DNMT1, located next to the H4K20me3-binding pocket of the BAH1 domain (Supplementary Fig. 8b), is reportedly linked to schizophrenia^44^. How the disease-associated DNMT1 BAH1 mutations affect genomic methylation and disease progression awaits further investigation.

While epigenetic silencing of repeated elements such as endogenous retroviruses (ERVs) and LINE-1 elements is crucial for maintaining integrity of the mammalian genome, the underlying silencing mechanisms are rather complex. Previous studies have pointed to a set of repressive complexes involved in H3K9me3^45–47^ or DNA methylation^48,49^ pathways. In this work, we uncovered that the interaction between the BAH1 domain of DNMT1 and H4K20me3 is especially important for optimal DNA methylation at the 5′ *cis*-regulatory region of the newly evolved, non-truncated LINE-1 subfamily. A recent study also reported the repression of evolutionarily young LINE-1 by N^6^-methyladenine^50^. It is conceivable that complicated interplays exist among H3K9me3, H4K20me3, and DNA methylation by DNMT1 along with cofactors^29^ and DNMT3A/3B (in complex with DNMT3L^51,52^). Further investigation is warranted to determine the relative contribution of these epigenetic pathways to repression of different subgroups of ERVs and LINE-1 elements.

The specific interaction between DNMT1 BAH1 and H4K20me3 adds to the growing list of BAH domain-mediated histone readouts, which include the H4K20me2 readout by the BAH domain of ORC1^32^ and the H3K27me3 readout by the BAH domains of effector proteins in *Neurospora crassa*, plants and mammals^4,33–35^. Intriguingly, these BAH domains harbor a similar histone-binding surface, but with distinct binding modes (Supplementary Figs. 2b and 8c, d), highlighting the evolutionary divergence of the histone modification-binding mechanisms of this reader module family.

**Fig. 6:**
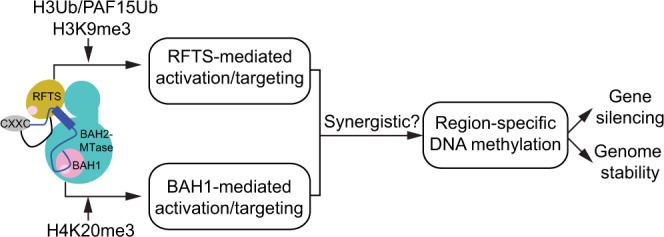
A model for DNMT1 RFTS- and BAH1-regulated DNA methylation. Structural elements of DNMT1, including RFTS, CXXC, autoinhibitory linker and BAH1 are colored in corn, gray, blue and pink, respectively. The BAH2 and MTase domains are colored in cyan. Histone marks (H3K9me3 and H4K20me3) cooperate with UHRF1-generated H3Ub or PAF15Ub in activation and targeting of DNMT1 for optimal DNA methylation at specific genomic loci, which in turn leads to gene repression and genomic stabilization. Note that the H3K9me3Ub and H4K20me3 marks likely regulate the activity of DNMT1 in a synergistic manner.

Second, this study delineates how DNMT1 transduces a multitude of environmental cues into its targeting and enzymatic activity. It has been established that Ubiquitin-like, containingPHD and RING Finger domains, 1 (UHRF1) plays a critical role in activating and targeting DNMT1 during S phase: UHRF1-mediated ubiquitylation of PAF15^26,27^ and histone H3^22–25^ serves to recruit DNMT1 to the replication foci during early and late S phase, respectively. This study demonstrates that the BAH1–H4K20me3 readout cooperates with the RFTS–H3K9me3/H3Ub readout in allosterically stimulating DNMT1, thereby uncovering another regulatory axis in DNA methylation maintenance in heterochromatin domains (Fig. 6). Disruption of these histone-directed regulations in cells impairs the CpG methylation by DNMT1, leading to an aberrant landscape of DNA methylation and defects in the maintenance of genome stability, a phenotype known to be associated with cancer^53^ and developmental disorders^54^. Note that neither RFTS- nor BAH1-mediated DNMT1 activation involves the discrimination of the methylation state of DNA substrates, providing a potential mechanism for compensating the “imprecise” maintenance methylation activity of DNMT1, thereby strengthening the region-specific methylation maintenance^13,55^. It is worth mentioning that maintenance of histone lysine (e.g., H3K9 and H4K20) methylation has been shown to be gradually established following S phase and may differ between parental and newly incorporated histones^56–58^. For instance, the H4K20 of recycled histones is dominantly methylated during replication, whereas the new histones only become methylated in the G2/M phase^56^. In this regard, it is likely that H4K20me3- and H3K9me3-mediated DNMT1 targeting and activation may be initiated by recycled parental nucleosomes and persist beyond S phase, providing a mechanism that cooperates with the S phase-specific, UHRF1-mediated regulation for efficient DNA methylation maintenance. Indeed, recent studies have indicated that the de novo methyltransferase activity of DNMT1, partially mediated by the BAH domains, is important in reinforcing DNA methylation maintenance in imprinting control or H3K9me3-marked regions^13,14^.

Together, this study uncovers a signaling pathway in which a BAH domain harbored within DNMT1 acts to transform H4K20me3-marked chromatin domains, in particular LINE-1 elements, into a more stable, DNA damage resistant, heterochromatic state with optimal levels of DNA methylation.

## Methods

### Plasmids

The DNMT1 cDNA was purchased from Addgene (cat # 24952). For cellular assays, full-length DNMT1 with an N-terminal 3×Flag tag was inserted into the pPyCAGIP vector^59^, a kind gift of I. Chambers. All of the DNMT1 point mutations were generated by a QuikChange II XL Site-Directed Mutagenesis Kit (Agilent). For all the in vitro assays, DNA encoding the human DNMT1 BAH1 domain (residues 728–900, hDNMT1_BAH1_), the bovine DNMT1 BAH1 domain (residues 725–897, bDNMT1_BAH1_), or the JMJD2A-Tudor domain (amino acids 894–1011, JMJD2A_TD_) was cloned into a modified pRSF-Duet vector, which introduced an N-terminal His_6_-SUMO tag and ULP1 (ubiquitin-like protease 1) cleavage site. To facilitate crystallization, residues 838–858 of bDNMT1_BAH1_ were replaced by a GAGSA sequence. For analysis of hDNMT1_351–1600_ methylation activity and H4K20me3 binding, the interaction between hDNMT1_BAH1_ and the autoinhibitory linker and the interaction between the hDNMT1 BAH2 domain (901–1107, hDNMT1_BAH2_) and H4K20me3, the hDNMT1 constructs were inserted into an in-house expression vector as a His_6_-MBP-tagged form. For peptide binding assay, hDNMT1_BAH1_, hDNMT1 fragment spanning the BAH1, BAH2, and MTase domains (residues 728–1600, hDNMT1_728–1600_) or JMJD2A_TD_ (amino acids 895–1011) was cloned into pGEX6P-1 vector. For fluorescence labeling, an hDNMT1 fragment (residues 351–639), containing C409S/C420S/S570C/C580S/T616C mutations and a C-terminal Lys-Pro-Glu-Thr-Gly tail, referred herein to as hDNMT1_RFTS-linker_, was also cloned into the modified pRSF-Duet vector. For sortase ligation with hDNMT1_RFTS-linker_, an hDNMT1 fragment (residues 646–1606) containing a C-terminal Lys-Pro-Glu-Thr-Gly tail was cloned into the in-house expression vector preceded by a His_6_-MBP-tag. All plasmid sequences were verified by sequencing before use, with the residue numerations based on the isoform 1 of DNMT1 that contains 1616 amino acids. The primers used for cloning are listed in Supplementary Table 2.

### Protein purification

The *E. coli* BL21(DE3) RIL cells (Novagen Inc) were used for protein expression. The cells harboring the expression plasmids were first cultured in LB medium at 37 °C until the OD_600_ (optical density at 600 nm) reached 0.6. Totally, 0.1 mM isopropyl β-d-1-thiogalactopyranoside (IPTG) was then added to induce the protein expression at 16 °C overnight. After harvesting, the cells were resuspended and lysed in a buffer containing 50 mM Tris-HCl (pH 7.5), 25 mM imidazole, 1 M NaCl, 0.5 mM DTT and 1 mM PMSF. To purify the His_6_-SUMO-tagged hDNMT1_BAH1_, bDNMT1_BAH1_ or JMJD2A_TD_ protein, the clarified supernatant was applied to a nickel column and the fusion protein was eluted with buffer containing 25 mM Tris-HCl (pH 8.0), 100 mM NaCl and 300 mM imidazole. The eluted protein was then subjected to tag removal by ULP1 cleavage, anion exchange chromatography on a HiTrap Q XL column (GE Healthcare), nickel affinity chromatography, and size-exclusion chromatography on a HiLoad 16/600 Superdex 75 pg column (GE Healthcare) that was pre-equilibrated with buffer containing 20 mM Tris-HCl (pH 7.5), 50 mM NaCl and 5 mM DTT. For ITC binding assay, human ORC1_BAH_ (residues 1–185) was purified as described previously^32^. The His_6_-MBP tagged DNMT1 proteins were sequentially purified via Ni^2+^ chromatography, ion-exchange chromatography on a Heparin HP (GE Healthcare) or Q HP column (GE Healthcare), TEV protease cleavage for tag removal, a second round of nickel affinity chromatography, and size-exclusion chromatography on a Superdex 200 16/600 column (GE Healthcare). The GST-tagged hDNMT1_BAH1_ and JMJD2A_TD_ for tri-methylated histone peptides-binding assay was purified through GST-affinity chromatography and eluted in 100 mM Tris-HCl (pH 8.0), 10 mg/mL reduced glutathione (Sigma-Aldrich). Purification of GST-hDNMT1_728–1600_ fusion protein also involved ion-exchange chromatography on a Heparin HP column and size exclusion chromatography on a HiLoad 16/600 Superdex 200 pg column (GE Healthcare). The final protein sample was stored in a buffer containing 20 mM Tris-HCl (pH 7.5), 250 mM NaCl, 5% Glycerol, and 5 mM DTT. DNMT1 mutants were introduced by site-directed mutagenesis and purified as that described for wild-type protein. For preparation of H3K9me3Ub2 peptide, the His_6_-SUMO-tagged Ub(G76C) protein was purified through Ni^2+^ chromatography, followed by tag removal via ULP1 cleavage and a second step of Ni^2+^ chromatography. All purified protein samples were stored at −80 °C before use.

### Chemical modification of histone peptide

The H3K9me3Ub2 peptide was generated and purified as previously described^29^, following a published protocol^37^. In essence, the H3_1–24_K9me3K18CK23C peptide was synthesized from LifeTein LLG, with an additional C-terminal tyrosine for spectroscopic quantification. To prepare the H3K9me3Ub2 peptide, the Ub(G76C) protein was mixed with the H3_1–24_K9me3K18CK23C peptide in a 4:1 molar ratio in buffer containing 250 mM Tris-HCl (pH 8.6), 8 M urea and 5 mM TCEP, and incubated at room temperature for 30 min. The cross-linker 1,3-dichloroacetone was then added to the reaction mixture with the amount equal to one-half of the total sulfhydryl groups. After 2 h-incubation on ice, the reaction was stopped by 5 mM β-Mercaptoethanol. The H3_1–24_K9me3Ub2 peptide was further purified using a mono S column (GE Healthcare). The caveat of this chemical modification method is that the crosslinking to be introduced is only an analog of the isopeptide bond between Ub and lysine.

### Crystallization and structure determination

To prepare the bDNMT1_BAH1_–H4_14–25_K20me3 and bDNMT1_BAH1_–H4_14–25_K20me2 complexes, the bDNMT1_BAH1_ protein was mixed with the H4_14–25_K20me3 or H4_14–25_K20me2 peptide, each containing an additional C-terminal tyrosine in a 1:3 molar ratio, and incubated on ice for 30 min. The bDNMT1_BAH1_–H4_14–25_K20me3 and bDNMT1_BAH1_–H4_14–25_K20me2 complexes were crystallized in a buffer containing 0.1 M HEPES (pH 7.5) (for bDNMT1_BAH1_–H4_14–25_K20me3) or 0.1 M MES (pH 6.5) (for bDNMT1_BAH1_–H4_14–25_K20me2), 13% PEG1500, 20 mM DTT, and 200 mM l-proline at 4 °C, using the hanging-drop vapor diffusion method. The crystals were soaked in the crystallization buffer supplemented with 20-25% (v/v) glycerol as cryo-protectant before flash frozen in liquid nitrogen. The X-ray diffraction data were collected on the beamline 24-ID-E, NE-CAT at Advanced Photo Source (APS) for bDNMT1_BAH1_–H4K20me3, and beamline BL92 at Stanford Synchrotron Radiation Lightsource (SSRL) for bDNMT1_BAH1_–H4K20me2. The data were indexed, integrated and scaled by HKL2000 program^60^ or XDS^61^. The structures were solved by molecular replacement using PHASER^62^ with the corresponding domain in the DNMT1 structure (PDB 4WXX [10.2210/pdb4WXX/pdb]) as searching model. Iterative cycles of model rebuilding and refinement were carried out using COOT^63^ and PHENIX^64^, respectively. Data collection and structure refinement statistics were summarized in Table 1.

### ITC binding assay

ITC measurements were performed at 5 °C using a MicroCal iTC200 instrument (GE Healthcare). The peptides, including hDNMT1_714-727_, H4_14–25_K20me0, H4_14–25_K20me1, H4_14–25_K20me2 and H4_14–__25_K20me3, were synthesized from LifeTein LLG, with an additional C-terminal tyrosine for spectroscopic quantification. To measure the bindings between the hDNMT1 BAH1 or BAH2 domain samples and H4K20me peptides, 1 mM H4K20me peptide was titrated over 0.1 mM hDNMT1_BAH1_ or hDNMT1_BAH2_ in buffer containing 25 mM Tris-HCl (pH 7.5), 100 mM NaCl and 2 mM fresh DTT. To measure the bindings between MBP-tagged hDNMT1_351–1600_ proteins and H4_14–25_K20me3, 1.44 mM peptide was titrated over 72 μM MBP-hDNMT1_351–1600_ in buffer containing 20 mM Tris-HCl (pH 7.5), 100 mM NaCl and 1 mM DTT at 5 °C. The spacing between injections was set to 180 s. To measure the binding between ORC1 BAH domain and H4K20me2 peptide, 0.45 mM peptide was titrated over 64 μM ORC1 BAH domain in buffer containing 20 mM Tris-HCl (pH 7.5), 100 mM NaCl and 1 mM fresh DTT. Analyses of all data were performed with MicroCal Origin software, fitted with single-site binding mode. The ITC binding parameters are summarized in Supplementary Table 1.

### Histone peptide microarrays and pulldowns

Histone peptide microarrays and in-solution peptide pulldowns were performed as described previously^32,65–67^. To evaluate the bindings of DNMT1 to H4 peptides with different K20 methylation states, 25 pmol of the GST-hDNMT1_728–1600_ was incubated with 250 pmol of biotinylated H4_11–27_K4me0, H4_11–27_K20me1, H4_11–27_K20me2, and H4_11–27_K20me3 peptide for 1 h. with rotation at 4 °C in Peptide Pulldown Buffer (50 mM Tris, 0.1% NP-40, 0.5% bovine serum albumin (BSA), 150 mM NaCl, pH 8.0). After this, 2.5 µL of streptavidin magnetic beads that had been blocked in the same buffer were added to the reaction and allowed to incubate for an additional 1 h. Next, 3 × 5 min washes were performed using the same buffer. Protein was eluted with 1× Laemmli’s SDS Loading Buffer and ran for a Western blot using standard molecular biology techniques. The input lanes represent 1% of the total protein loaded per reaction and each lane account for 10% of the total reaction. The primary antibody used was anti-GST at 1:20,000 (EpiCypher, 13-0022) and the secondary was anti-Rabbit-HRP at 1:20,000 (GE, NA934V). Imaging was performed using a chemiluminescent kit (GE, RPN2232) and BioRad ChemiDoc system. To evaluate the bindings of hDNMT1_BAH1_ and JMJD2A tudor domain (JMJD2A_TD_) to various tri-methylated histone peptides, H3_1–__21_K4me3, H3_1–21_K9me3, H3_1–21_K14me3, H3_10–27_K18me3, H3_21–44_K27me3, K3_21–44_K36me3, and H4_11–29_K20me3 peptides were synthesized as previously described^68^. One microgram of peptide was incubated with 2 µg of recombinant proteins in the binding buffer containing 50 mM Tris-HCl (pH 7.5), 150 mM NaCl, and 0.1% NP-40. Peptides were pulled down using streptavidin sepharose beads (Amersham) and protein–peptide bindings were detected by western analyses using anti-GST at 1:2500 (custom rabbit polyclonal)^66^ and anti-Rabbit-HRP at 1:10,000 (Cell Signaling Technologies).

### DNA methylation kinetics assay

The DNA methylation assays were performed as previously described^29^. In essence, each reaction mixture contains 0.1 μM hDNMT1_351–1600_, wild type or mutants, 0.5 μM S-adenosyl-l-[methyl-^3^H] methionine (SAM) (Perkin Elmer), 0.4 μM (GT^m^C)_12_/(GAC)_12_ hemimethylated DNA duplex, or various amount of histone peptides in 50 mM Tris-HCl (pH 8.0), 7 mM β-ME, 5% glycerol, 100 μg/mL BSA, and 100 mM NaCl. Each reaction lasted 20 min at 37 °C, before being quenched by 2 mM cold SAM. The activity was measured by Beckman LS6500 scintillation counter.

### Single-molecule Förster resonance energy transfer (smFRET) experiment

The DNMT1 constructs used for the smFRET experiment was prepared by sortase-mediated ligation of hDNMT1_RFTS-linker_, labeled with both Cy3 (GE Healthcare) and Atto647N (Sigma-Aldrich), with the C-terminally biotin-conjugated hDNMT1_646–1606_-LPETG fragment. In detail, the hDNMT1_RFTS-linker_ sample was reduced by 10-fold molar excess of TCEP in buffer containing 20 mM Tris-HCl (pH 7.3), 150 mM NaCl and 5% glycerol for over 30 min, before reacting with tenfold molar excess of Cy3 maleimide and fivefold molar excess of Atto 647N maleimide, first at room temperature for 2 h, then at 4 °C overnight in darkness. The unreacted dye was subsequently removed through desalting using a PD-10 column (GE healthcare). Meanwhile, the hDNMT1_646–1606_-LPETG sample was incubated with tenfold molar excess of GGGK^biotin^ (K^biotin^: biotinylated lysine) peptide and twofold molar excess of sortase in ligation buffer (20 mM Tris-HCl (pH7.3), 150 mM NaCl, 5 mM CaCl_2_, 5% glycerol) at room temperature for 4 h, followed by removal of unreacted peptide using a PD-10 column. Both protein samples were stored in buffer containing 20 mM Tris-HCl (pH7.5), 150 mM NaCl, 5% glycerol and1 mM β-Mercaptoethanol. Next, ~10 μM Cy3- and Atto647N-labeled hDNMT1_RFTS-linker_ was incubated equimolar with biotinylated hDNMT1_646–1606_-LPETG and sortase in the ligation buffer at room temperature for 2 h. The ligated hDNMT1 product was further purified on a HiLoad 16/600 Superdex 200 pg column (GE healthcare). The protein homogeneity was verified by sodium dodecyl sulphate-polyacrylamide gel electrophoresis (SDS-PAGE). The protein concentration and the efficiency of dye incorporation were measured using NanoDrop (Thermo Fisher Scientific).

The smFRET experiments were performed at 20 °C. A commercial, biotinylated Pacific Biosciences SMRT cell was incubated with NeutrAvidin (25 µM) (Thermo Fisher Scientific) for 5 min, then unbound NeutrAvidin was washed away. Twenty microlitres of 10 nM hDNMT1_RFTS-linker_–hDNMT1_646–1606_–LPETG ligated sample (hDNMT1^CY^) was then surface-immoblized by incubation for 5 min with the NeutrAvidinylated cell, followed by further washing with buffer (20 mM Tris-HCl (pH7.5), 150 mM NaCl, 5% glycerol, 1 mM β-Mercaptoethanol) to remove unbound protein. During the final wash, oxygen-scavenging buffer containing 2.5 mM protocatechuic (PCA), 250 nM protocatechuate-3,4-dioxygenase (PCD), 2 mM triplet quencher mixture (TSY, Pacific Biosciences)) were included^69,70^. The SMRT cell with immobilized protein was then placed in the RS II instrument (Pacific Biosciences) and imaged by illumination with a 532-nm laser (0.7 µW/m^2^). For experiments with histone peptides, the peptide was robotically delivered to the SMRT cell by the instrument at the beginning of the movie. Movies were recorded for 10 min at 10 fps. The delivery mix was made of the final wash buffer supplemented with 2–6 mM H4_11–25_K20me3 (containing a C-terminal tyrosine) or 100 nM H3_1–24_K9me3Ub2 or 2 mM H3_1–15_K4me3 (containing a C-terminal tyrosine).

### smFRET data analysis

Data processing were performed with in-house MATLAB scripts^70^. Individual single-molecule fluorescence traces were extracted from raw movie data, filtering for those that showed colocalized green and red fluorescence. A substantial fraction of the molecules exhibited significant fluorophore photophysical instability, with significant excursions in the donor intensity on a seconds time scale, including transient periods of complete donor quenching. To minimize the possibility of artifacts, traces were therefore curated manually and their event timings were assigned manually. Traces for assignment were thus selected with the following characteristics: (1) The beginning of all assigned FRET events was either the beginning of the movie or a frame in which there was an abrupt increase in the red signal with an anticorrelated decrease in the green signal, and (2) the end of all assigned events was an abrupt decrease in the red signal with an anticorrelated increase in the green signal. The resulting FRET event time durations for each condition were then converted to an empirical cumulative distribution function and fit to a single-exponential (*P*(*t*) = 1 − e^–*λt*^) or double-exponential $(p(t)=A(1-{e^{-{\lambda_{1}}t}})\ +\ (1-{\mathrm{{A}}})(1-{e^{-{\lambda_{2}}t}}))$ model as required to maximize the *R*^2^ value from nonlinear least-squares regression.

### MD simulations

To probe the effect of the H4K20me3 binding and the W796A mutation on the conformation and flexibility of the autoinhibitory linker of hDNMT1, MD simulations were carried out for WT and the W796A mutant of hDNMT1_351–1600_, and a model for the hDNMT1_351–1600_–H4_14–25_K20me3 complex using the OpenMM software package on GPUs. Two independent trajectories were conducted for the complex. As the starting conformation, the crystal structure of hDNMT1_351–1600_ (PDB 4WXX [10.2210/pdb4WXX/pdb]) was used for the simulation of WT and the W796A mutant; for the hDNMT1_351–1600_–H4_14–25_ K20me3 complex, a structural model was generated based on the structural alignment of hDNMT1_351–1600_ with the bDNMT1_BAH1_–H4_14–25_K20me3 complex, with the conformation of residues 712–729 of hDNMT1_351-1600_ adjusted using the coot software^63^ to accommodate the H4_14–25_K20me3 binding. For each MD simulation, the protein was solvated using a cubic water box such that the protein was at least ten Angstroms away from the box edge. The system was charge neutralized with NaCl counter ions and a salt concentration of 150 mM was maintained during the simulation. The system contained approximately 374,000 atoms. For each system, equilibration of 50 ps was done with the NVT ensemble, while production run of 100 ns was conducted with the NPT ensemble using an isotropic Monte Carlo barostat, a Langevin thermostat and an integration time step of 2 fs. For non-bonded interactions, a force-switch based scheme (r_on = 1.0 nm, r_off = 1.2 nm) was used for van der Waals interactions, and Particle–Mesh–Ewald summation with a grid size of approximately 1 Å was used for treating electrostatic interactions. For analysis, structures were saved every 10 ps. For the computation of root-mean-square-fluctuations, only the last 20 ns of the 100 ns production run was used.

### Cell lines and tissue culture

The murine Dnmt1-knockout (1KO) ESCs were used as we previously described^29^. In brief, 1KO-ESCs were transfected with the pPyCAGIP empty vector or that carrying WT or mutant DNMT1, followed by drug selection with 1 μg/mL puromycin for over two weeks. Both the pooled stable-expression lines and independent single-cell-derived clonal lines were established and verified (such as immunoblotting of DNMT1) before use.

### Antibodies and Western blotting

The cultured cells were collected, rinsed in cold phosphate-buffered saline (PBS), and suspended in buffer containing 50 mM Tris-HCl (pH 8.0), 150 mM NaCl and 1% NP-40. After brief sonication and centrifugation, the soluble fractions of cell lysates were mixed with 2× SDS-PAGE loading buffer and boiled for 5 min, followed by loading onto a SDS-PAGE gel for immunoblotting analysis, as previously described^51,71^. Information for the antibodies used in this work is included in the Supplementary Table 3.

### Quantification of 5-methyl-2′-deoxycytidine (5-mdC) in gDNA

Measurements of the global levels of 5-mdC in cellular DNA were carried out as described previously^29,51^. Briefly, gDNA was digested into mononucleosides by nuclease P1 and alkaline phosphatase. The enzymes in the digestion mixtures were removed by chloroform extraction, and the resulting aqueous layer was dried, reconstituted in water, and subjected to LC–MS/MS/MS analyses on an LTQ XL linear ion trap mass spectrometer for quantifications of 5-mdC and dG. The amounts of 5-mdC and dG (in moles) in the nucleoside mixtures were calculated based on comparisons of their signal intensities with their corresponding stable isotope-labeled standards and calibration curves. The final levels of 5-mdC were calculated as molar ratios of 5-mdC over dG.

### Confocal immunofluorescence (IF)

G1/S phase synchronization of ES cells follows a previously established protocol^29^. In essence, ES cells were treated with thymidine (Sigma T9250) at a final concentration of 2 mM for 16 h, washed twice with prewarmed PBS, and then grown in fresh ES cell medium. After a 6 h release, thymidine was added to the medium again. Cells were incubated with thymidine for another 16 h, washed, released for 5 h from the thymidine block with the addition of fresh medium and then proceed for IF. The IF was carried out as described before^29,72^.

### Co-immunoprecipitation (CoIP)

Cells were lysed as described^29,72^. Anti-Flag M2-conjugated agarose beads (Sigma) were incubated with the lysates overnight at 4 °C. The beads were then extensively washed and the bound proteins analyzed by western blotting.

### Enhanced reduced representation bisulfite sequencing (eRRBS)

The eRRBS experiment, including construction of libraries and processing and analysis of eRRBS dataset, was performed as described previously^29^. Briefly, genomic DNA (gDNA) mixed with 0.1% of unmethylated lambda DNA (Promega) was digested with MspI, MseI, and BfaI, and subjected to end repair, A-tailing and ligation to NEBNext Methylated Adapters (NEBNext DNA Library Prep Kit). Next, the DNA product was purified using AMPure beads and subject to bisulfite conversion and library construction using the EpiMark Bisulfite Conversion Kit (NEB cat# E3318), followed by deep sequencing in an Illumina HiSeq 4000 platform with a paired end PE150 cycle (carried out by UNC HTSF Genomic Core). The eRRBS data were then analyzed with FastQC v0.11.2 [http://www.bioinformatics.babraham.ac.uk/projects/fastqc/] and Bismark v0.18.1^73^. Analysis of the sequences corresponding to phase λ indicated a bisulfite conversion rate of >99%.

### DMR detection

Counts of methylated and unmethylated bases for cytosines in the CpG context were analyzed with the DSS (v.2.30.1) R/Bioconductor package^74^ for the calling of differential methylation loci or regions (DML/DMRs) and their quantification. The *q* values under 0.05 and mean differences of methylation greater than 0.15 were used as the cutoff line to define DMRs with DSS. Alternative identification of DML/DMRs was also performed using the R-package methylKit^75^ (v.1.8.1) with tiling windows of 500 bp. In the setting of this tool, *q* values under 0.01 and mean differences of methylation greater than 0.25 were used as the cutoff line. In both analyses, at least five covered cytosine sites were utilized. After the calling of differential methylation, each locus or region was annotated using Homer^76^ (v4.10.3).

### ChIP-seq and data analysis

ChIP-seq data of H3K9me3 in the E14Tg2a.4 mouse ES cell line was obtained from ENCODE for both raw FASTQ files (ENCODE ENCFF001ZHD, and ENCFF001ZHF) and called peaks (ENCODE ENCFF180LQA). ChIP-seq data of H4K20me3 in the R1 mouse ES cell line was obtained from GEO GSE94086^77^ as raw FASTQ files (SRA SRR5198791, SRR5198793). Sequence data was processed as follows: mapping to mm10 via Bowtie2 v2.1.0^78^ (parameters –sensitive-local), deduplication via Picard tools v1.110 MarkDuplicates.jar [http://broadinstitute.github.io/picard], then extension of each mapped non-duplicate read to assumed fragment length (200 nt for H3K9me3, 150 nt for H4K20me3). Depth tracks for the ChIP-seq data were generated by BEDTools v2.24.0 ‘genomecov’, followed by bedGraphToBigWig (UCSC utility script; http://hgdownload.soe.ucsc.edu/admin/exe/). Peak calls for H4K20me3 were made via HOMER findPeaks (parameters -region -size 1000 -minDist 2500). Peak sets were filtered to only include peaks on canonical chromosomes (chr1–19,X,Y), resulting in final peak sets of *N* = 123,309 (H3K9me3) and *N* = 24,373 (H4K20me3). Peaks were assigned to quartiles of decreasing ChIP-seq signal according to RPKM per peak region, which was calculated based on overlapping read counts as reported by BEDTools v2.24.0 ‘multicov’. Random genomic regions size-matched to each peak set were generated by BEDTools v2.24.0 [see reference above] ‘shuffle’ with parameters -noOverlapping -excl (where the excluded regions are defined as long poly-N stretches and all non-canonical chromosomes). For plotting of ChIP-seq signals, each signal was log2 transformed after addition of 0.01 as pseudo values.

### CUT&RUN followed by deep sequencing

CUT&RUN were performed according to manufacturer’s instructions (EpiCypher CUTANA^™^ pAG-MNase for ChIC/CUT&RUN, Cat# 15-1116). In brief, after washing with CUT&RUN wash buffer (20 mM HEPES pH 7.5, 150 mM NaCl, 0.5 mM spermidine, 1× Roche Complete Protease Inhibitor), a million of cells were first bound to activated ConA beads (Bangs Laboratories, cat# BP531), followed by addition of anti-H4K20me3 antibody (Abcam, ab9053; 1:100 dilution) and cell permeabilization with the digitonin buffer (CUT&RUN wash buffer plus 0.01% digitonin). After washing in the digitonin buffer, samples were incubated with pAG-MNase, followed by additional washes with digitonin buffer. After the final wash, pAG-MNase activation was induced for DNA digestion by suspending cell samples in the pAG-MNase digestion buffer (digitonin buffer plus 2 mM CaCl_2_) and incubation on nutator at 4 °C for 2 h. Solubilized chromatin was released using the stop buffer (340 mM NaCl, 20 mM EDTA, 4 mM EGTA, 50 µg/ml RNase A, 50 µg/ml glycogen) and collected using a PCR cleanup kit (New England BioLabs [NEB] Monarch PCR & DNA Cleanup Kit, cat# T1030). Ten nanogram of the purified CUT&RUN-enriched DNA was used for preparation of multiplexed Illumina libraries using the NEB Ultra II DNA Library Prep Kit according to manufacturer’s instructions (NEB cat#E7103).

### CUT&RUN data analysis

Raw fastq data were mapped to the reference genome (mm10) using bowtie2.3.5^79^. The non-primary alignment, PCR duplicates, or blacklist regions from aligned data were removed by Samtools (v1.9), Picard MarkDuplicates funtion (ver 2.20.4), and bedtools (v2.28.0), respectively. Peak calling was performed using MACS2 (macs2 callpeak -f BAMPE -g hs/mm –keep-dup 1)^80^. Deeptools (v3.3.0) was used to make bigwig files, heatmaps, and averaged plotting of CUT&RUN signal^81^. Genomic binding profiles were generated using the deepTools “bamCompare” functions. The peaks were annotated in the reference genome (NCBI Reference Sequences [RefSeq]) and then used for heatmap and averaged signal plotting.

### CRISPR/Cas9-based genome editing for site-specifically mutating Dnmt1_BAH1_

As we recently described^4^, the Alt-R^®^ CRISPR–Cas9 System, together with a single-stranded oligodeoxynucleotide donor (ssODN), was employed to introduce a W799A point mutation to mouse Dnmt1 gene (equivalent to W796A in the human DNMT1 BAH1 domain) in E14 ESCs. In brief, trans-activating CRISPR RNA (tracrRNA) with ATTO^™^ 550, the S.p. HiFi Cas9 Nuclease V3, Electroporation Enhancer, and crRNA targeting the Dnmt1 genomic site to be mutated (with the sequence information of crRNA listed in Supplementary Table 2) were all ordered through IDT Inc. The ssODN was custom-designed with a tool of benchling ([https://benchling.com/crispr], the sequence information of ssODN listed in Supplementary Table 2) and synthesized as PAGE-purified Ultramer^®^ DNA oligonucleotides from IDT Inc. Three phosphorothioate bonds were added at both ends of ssODN to stabilize the donor oligo and make homology-directed repair more efficient. For introducing a Dnmt1 W799A mutation, the codon was mutated from TGG to GCG, which generates a site of MluI enzyme and thus facilitates subsequent cell screening and genotyping by enzyme digestion of PCR products. To make the crRNA:tracrRNA duplex, the crRNA and tracrRNA were mixed in equimolar concentration, followed by heating at 95 °C for 5 min and cooling down to room temperature. The CRISPR–Cas9 ribonucleoprotein (RNP) complex was then prepared by diluting the crRNA:tracrRNA duplex and Cas9 enzyme components in PBS, followed by incubation at room temperature for 15 min. The RNP complex was next mixed with Electroporation Enhancer and ssODN, followed by electroporation-based delivery to E14 cells using the Mouse Embryonic Stem Cell Nucleofector Kit (Lonza). ATTO^™^ 550 positive cells were sorted out by FACS (at UNC flow core) after 36 h and split into 96-well plates for genotyping. After genotyping, lines with homozygous mutation were further validated at DNA levels by direct sequencing of PCR products (the sequence information of genotyping primers listed in Supplementary Table 2).

### Chemical compound

The chemical inhibitor selective for the H4K20 methyltransferases SUV420H1 and SUV420H2, A-196^42^ (Sigma, SML1565), was dissolved in dimethylsulfoxide (DMSO) as 5 mM stock solution. A 3-day treatment with 10 µM A-196, compared to DMSO, was used in this study.

### Neutral comet assay

To perform neutral comet assays for DNMT1 C1226S mutants (Supplementary Fig. 7f–h), mouse DNMT1 knockout cells (7 × 10^4^ cells) complemented by pPyCAGIP empty vector (vector), WT DNMT1, and two different clones of DNMT1 C1226S mutant (clones #1 and #2) were plated on gelatin-coated culture dishes without feeder cells. After 2 h post ionizing irradiation (5 Gy) cells were harvested and mixed with LMAgarose (Trevigen). The LMAgarose mixed samples were placed onto comet assay slides and immerged into comet assay lysis solution (Trevigen) at 4 °C for 1 h. Subsequently, the slides were incubated with TBE buffer (90 mM Tris borate) for 1 h and subjected to electrophoresis at 40 V for 40 min. After electrophoresis, the samples were fixed with 70% ethanol at RT for 30 min and dried at 37 °C for 30 min. DNAs were visualized using SYBR-green (Invitrogen) and imaged using Fluoview FV3000 confocal microscope (Olympus). Images were subsequently analyzed using ImageJ (v.1.53). Statistics and graph were calculated using Prism software (Graphpad v6). Experiments were performed with at least two independent replicates. Neutral comet assays for cells with DNMT1 W465A, W465A/W796A, W464A/W465A, or TM (Fig. 5e, f), were performed as described previously^29^.

### Clonogenic cell survival assay

Cells were seeded into six-well plates and treated with different dosages of IR using a Faxitron X-ray irradiator. Following IR treatment, cells were incubated for 12 days in tissue culture incubator (37 °C, 5% CO_2_). Cells were washed with PBS and the colonies were stained with 0.5% (w/v) crystal violet and 20% (v/v) ethanol for 30 min at RT. Results were normalized to plating efficiencies of untreated cells for each group.

### Statistics

The comet assays were performed using one-way ANOVA with post Tukey analysis. The *p* value lower than 0.05 was considered to be statistically significant. For all the other analyses, the two-tailed Student *t* tests were performed to compare distributions between different groups. And the *p* value lower than 0.01 was considered to be statistically significant.

### Reporting summary

Further information on research design is available in the Nature Research Reporting Summary linked to this article.

## Supplementary information

Supplementary Information

Reporting Summary

Description of Additional Supplementary Files

Supplementary Data 1

Supplementary Data 2

Supplementary Data 3

Supplementary Data 4

## Data Availability

The data that support this study are available from the corresponding author upon reasonable request. Coordinates and structure factors for the bDNMT1_BAH1_–H4_14–25_K20me3 and bDNMT1_BAH1_–H4_14–25_K20me2 complexes have been deposited in the Protein Data Bank under accession codes 7LMK [https://doi.org/10.2210/pdb7LMK/pdb] and 7LMM [https://doi.org/10.2210/pdb7LMM/pdb], respectively. The eRRBS data generated during the course of this study have been deposited in Gene Expression Omnibus (GEO) under accession code GSE145698. Publically available datasets analyzed for this study: ChIP-seq data of H3K9me3 in the E14Tg2a.4 mouse ES cell line was obtained from ENCODE for both raw FASTQ files (ENCODE ENCFF001ZHD and ENCFF001ZHF) and called peaks (ENCODE ENCFF180LQA). ChIP-seq data of H4K20me3 in the R1 mouse ES cell line was obtained from GEO GSE94086 as raw FASTQ files (SRA SRR5198791 and SRR5198793). Source data are provided with this paper.

## Source data

Source Data
