# Supplementary material for: DNMT1 reads heterochromatic H4K20me3 to reinforce LINE-1 DNA methylation
Source: Nat Commun. 2021 May 3;12:2490. doi: 10.1038/s41467-021-22665-4 (PMC8093215; doi:10.1038/s41467-021-22665-4)
Supplement: Supplementary file 1 — Supplementary Information [file 41467_2021_22665_MOESM1_ESM.pdf]

Supplementary information

DNMT1 reads heterochromatic H4K20me3 to reinforce LINE-1 DNA methylation

Wendan Ren, Huitao Fan, Sara A Grimm, Jae Jin Kim, Linhui Li, Yiran Guo, Christopher James Petell, Xiao-Feng Tan, Zhi-Min Zhang, John P. Coan, Jiekai Yin, Dae In Kim, Linfeng Gao, Ling Cai, Nelli Khudaverdyan, Burak Çetin, Dinshaw J. Patel, Qiang Cui, Brian D. Strahl, Yinsheng Wang, Or Gozani, Kyle M. Miller, Seán E. O'Leary, Paul A. Wade, Gang Greg Wang, Jikui Song

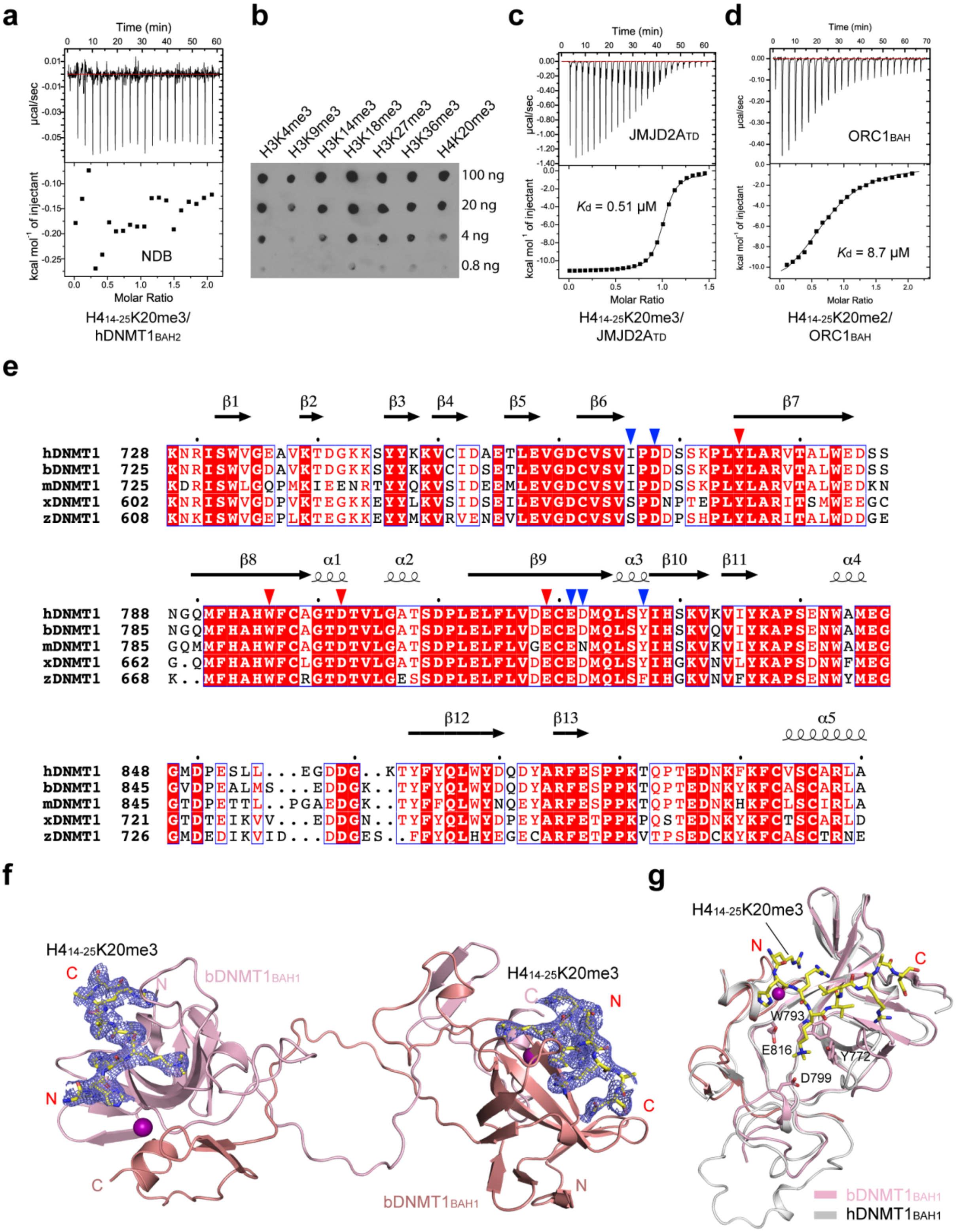

Supplementary Figure 1. Biochemical, sequence and structural analyses of the histone binding affinities of the DNMT1 BAH domains. (**a**) ITC binding curve for hDNMT1$_{BAH2}$ titrated with the H4$_{14\text{-}25}$K20me3 peptide. (**b**) Dot plot showing relative concentrations of biotinylated histone peptides used in peptide pulldowns detected

with peroxidase-conjugated streptavidin in Fig. 1e. (**c**) ITC binding curve for $JMJD2A_{TD}$ titrated with the $H4_{14\text{-}25}$K20me3 peptide. (**d**) ITC binding curve for $ORC1_{BAH}$ titrated with the $H4_{14\text{-}25}$K20me2 peptide. (**e**) Sequence alignment of the DNMT1 BAH1 domain from human (hDNMT1), bovine (bDNMT1), mouse (mDNMT1), Xenopus Laevis (xDNMT1) and Zebrafish (zDNMT1). Strictly conserved residues are colored in white and shaded in red. Similar residues are colored in red. The secondary structures corresponding to hDNMT1 BAH1 are marked on top. The residues forming the H4K20me3-binding pocket are indicated by red arrows. All the other H4-interacting residues are indicated by blue arrows. (**f**) Structure of two $bDNMT1_{BAH1}$-$H_{14\text{-}25}$K20me3 complexes engaging domain swapping. Due to crystal packing, the last two β-strands of $bDNMT1_{BAH1}$ were swapped with their counterparts from a crystal symmetry-related molecule, resulting in formation of a homodimer in crystals. The two BAH1 domains are colored in light pink and salmon respectively. The $H4_{14\text{-}25}$K20me3 peptide is shown as yellow sticks. The Fo-Fc omit map (blue) of the $H4_{14\text{-}25}$K20me3 peptide was contoured at the 2.0 σ level. The zinc ions are shown as purple spheres. (**g**) Structural superposition of $hDNMT1_{BAH1}$ (derived from PDB 4WXX) and the $bDNMT1_{BAH1}$-$H4_{14\text{-}25}$K20me3 complex. The structural overlay of $bDNMT1_{BAH1}$ and (PDB 4WXX) yields an RMSD of 1.0 Å over 130 Cα atoms.

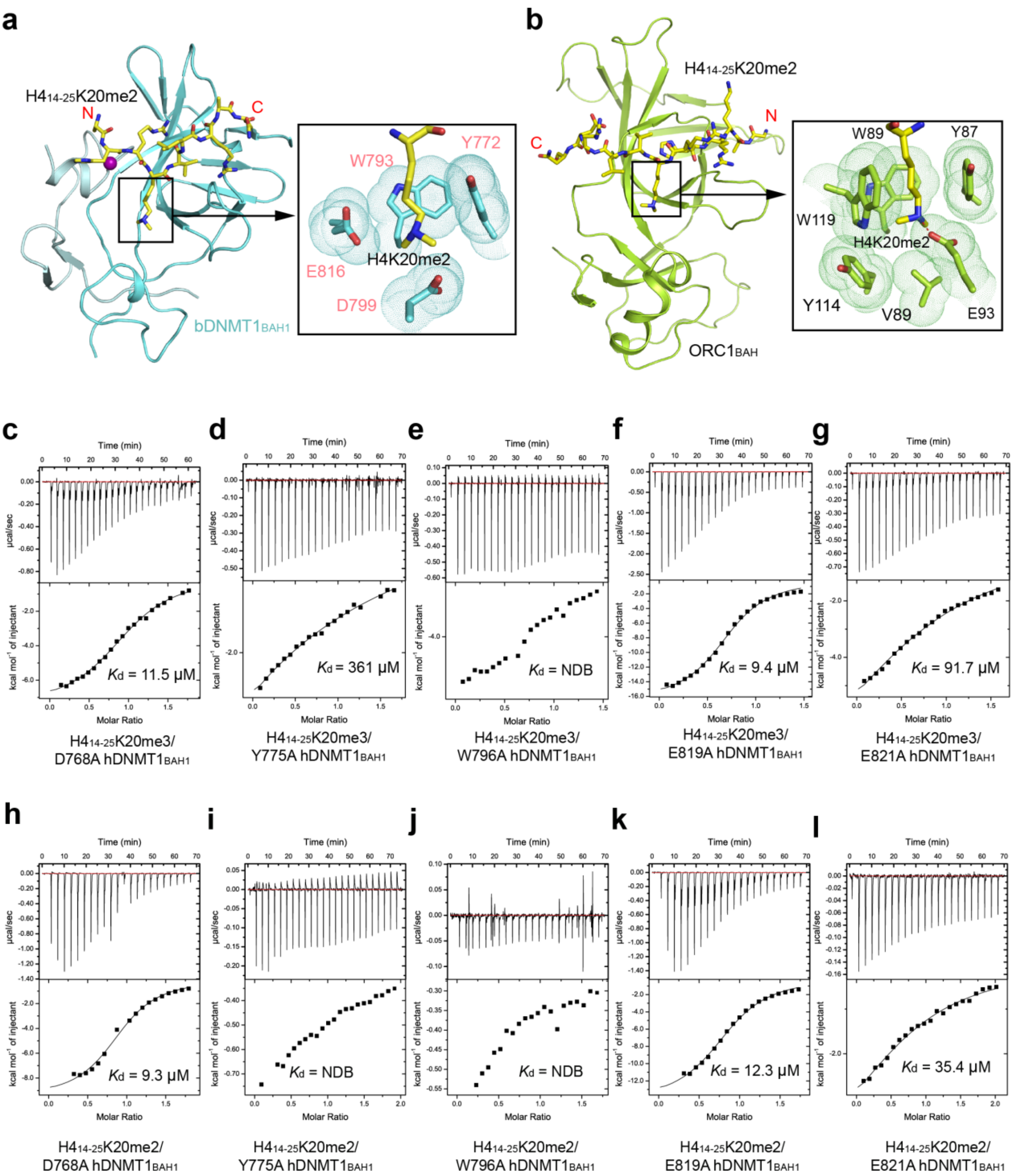

Supplementary Figure 2. Structural and biochemical analysis of the interaction between DNMT1 BAH1 domain and H4K20me2/3. (**a**) The crystal structure of $bDNMT1_{BAH1}$ (cyan) in complex with the $H4_{14\text{-}25}K20me2$ (yellow) peptide, with the H4K20me2-binding pocket shown in expanded view. (**b**) The crystal structure of $ORC1_{BAH}$ (green) in complex with the $H4_{14\text{-}25}K20me2$ peptide (yellow) (PDB 4DOW), with the H4K20me2-binding pocket shown in expanded view. (**c-g**) ITC binding curves of $hDNMT1_{BAH1}$ mutants with the $H4_{14\text{-}25}K20me3$ peptide. NDB: no detectable

binding. (**h-l**) ITC binding curves of $hDNMT1_{BAH1}$ mutants with the $H4_{14\text{-}25}K20me2$ peptide. NDB: no detectable binding.

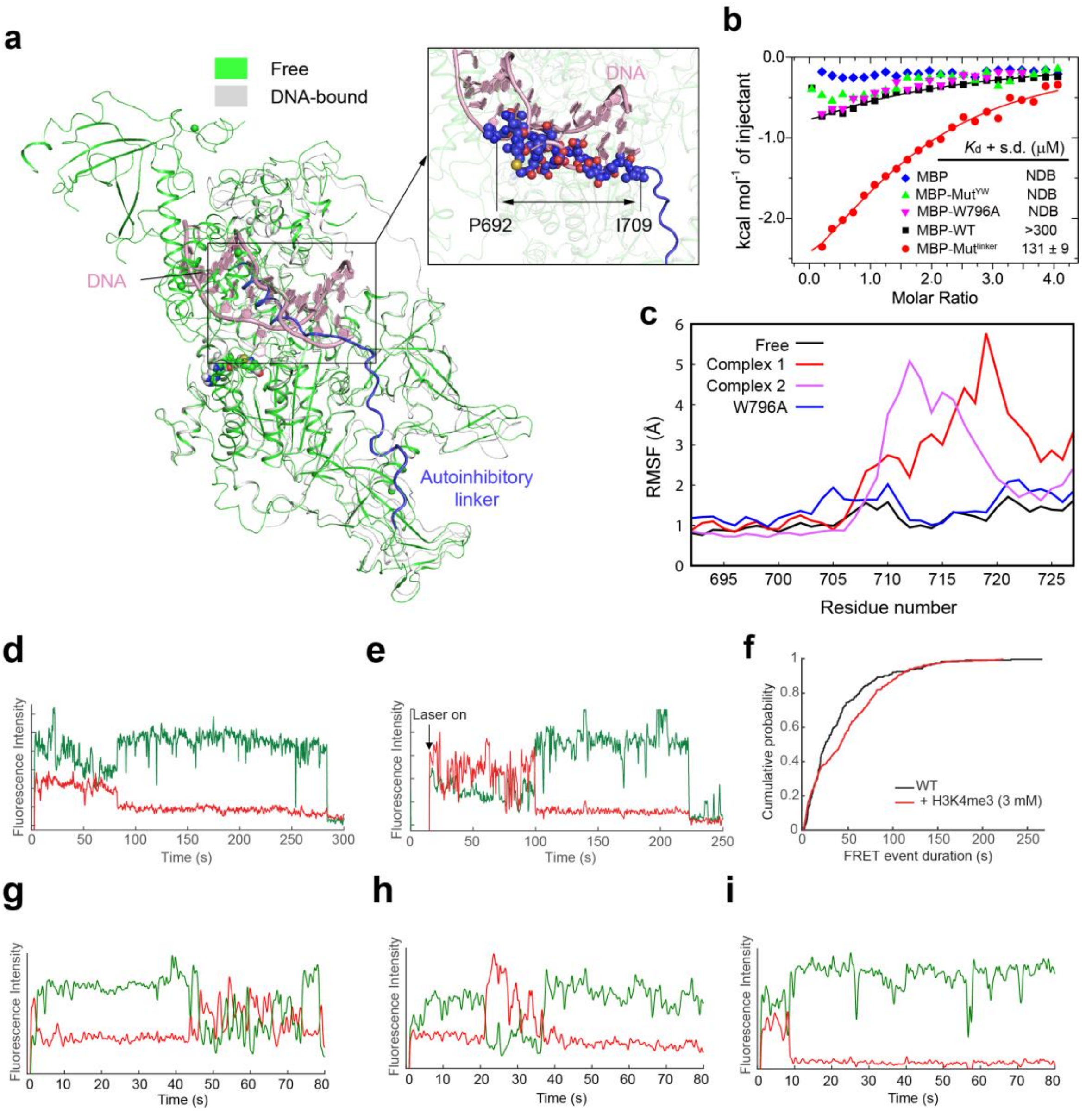

Supplementary Figure 3. Displacement of the autoinhibitory linker by the DNA or H4K20me3 binding of DNMT1. (**a**) Structural overlay DNA-free hDNMT1$_{351-1600}$ (PDB 4WXX; green) and the mDNMT1$_{731-1602}$-DNA complex (PDB 4DA4; grey). The autoinhibitory linker of hDNMT1$_{351-1600}$ is colored in blue. The mDNMT1$_{731-1602}$-bound DNA duplex is colored in light pink and clashes with residues P692-I709 (blue spheres) on the autoinhibitory linker of hDNMT1$_{351-1600}$ in expanded view. Zinc ions and SAH molecules are shown in sphere representation. (**b**) ITC binding analysis of the interaction between MBP-tagged hDNMT1$_{351-1600}$, WT, W796A, Mut^{YW} or Mut^{linker}, and the H4$_{14-25}$K20me3 peptide. (**c**) The RMSF values obtained for the autoinhibitory linker in the structural models of WT (PDB 4WXX) or W796A-mutated hDNMT1$_{351-1600}$ and the hDNMT1$_{351-1600}$-H4K20me3 complex (complex 1 and complex 2) during 100 ns MD simulations. (**d,e**) Representative smFRET traces observed for hDNMT1^{CY} in the absence (d) or presence (e) of H3K4me3. (**f**) Cumulative probability of the

durations of FRET events observed for hDNMT1^{CY}, in the absence (black) or presence (red) of H3K4me3 peptide (212 and 151 molecules, respectively). (**g-i**) Representative smFRET traces observed for three population of peptide-free, W796A hDNTM1^{CY} showing different FRET states.

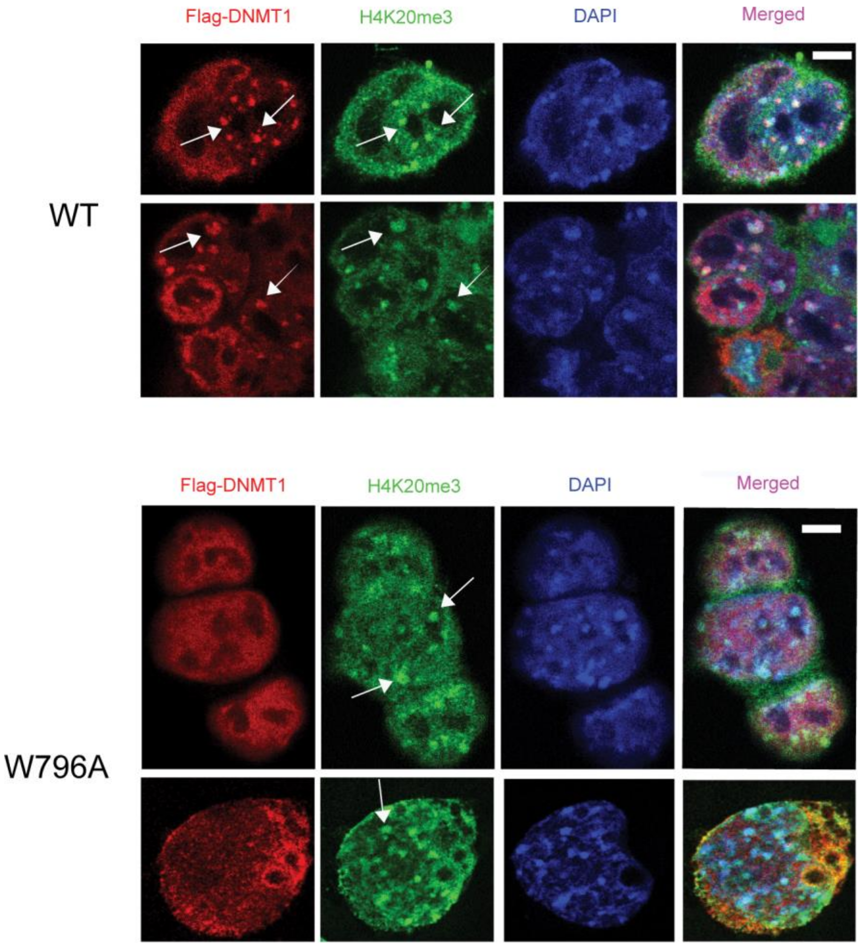

Supplementary Figure 4. Replicates of IF images in Fig. 4c. Representative confocal immunofluorescence images revealing localization of the indicated DNMT1 (Flag-tagged, red), H4K20me3 (green) and chromatin (stained by DAPI, blue) in the 1KO-ESC stable expression lines, synchronized in S phase. Scale bar, 5 micrometers. Selected H4K20me3 loci that are colocalized with WT (top) but not W796A (bottom) DNMT1 are indicated by arrows. The experiment was repeated 3 times with consistent results.

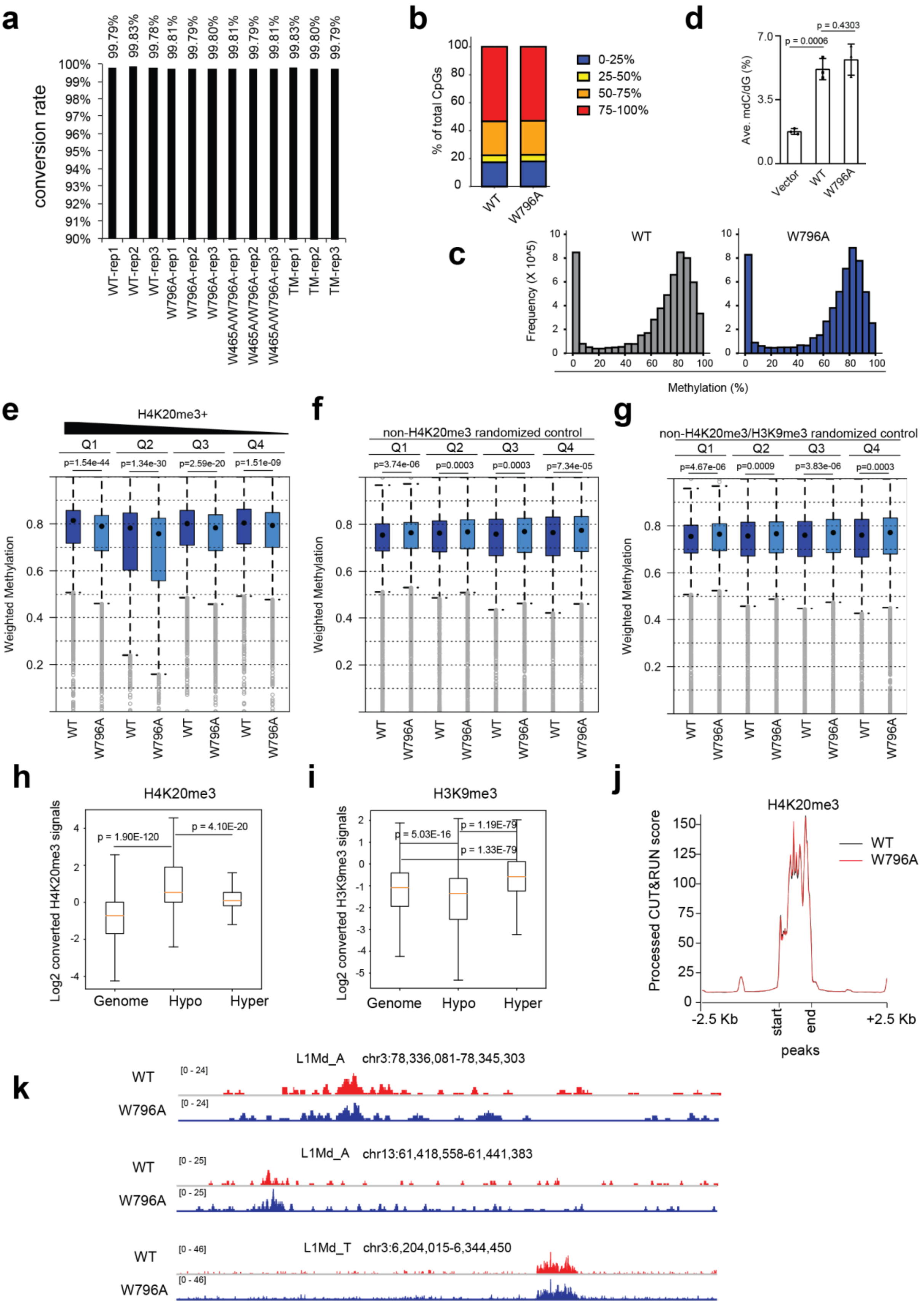

a
conversion rate
100%
99%
98%
97%
96%
95%
94%
93%
92%
91%
90%
99.79%
99.83%
99.78%
99.81%
99.79%
99.80%
99.81%
99.79%
99.81%
99.83%
99.80%
99.79%
WT-rep1
WT-rep2
WT-rep3
W796A-rep1
W796A-rep2
W796A-rep3
W465A/W796A-rep1
W465A/W796A-rep2
W465A/W796A-rep3
TM-rep1
TM-rep2
TM-rep3
b
% of total CpGs
0-25%
25-50%
50-75%
75-100%
WT
W796A
d
Ave. mdC/dG (%)
p = 0.0006
p = 0.4303
Vector
WT
W796A
c
Frequency (X 10^5)
WT
W796A
Methylation (%)
e
H4K20me3+
Q1
Q2
Q3
Q4
p=1.54e-44
p=1.34e-30
p=2.59e-20
p=1.51e-09
Weighted Methylation
f
non-H4K20me3 randomized control
p=3.74e-06
p=0.0003
p=0.0003
p=7.34e-05
g
non-H4K20me3/H3K9me3 randomized control
p=4.67e-06
p=0.0009
p=3.83e-06
p=0.0003
h
H4K20me3
Log2 converted H4K20me3 signals
p = 1.90E-120
p = 4.10E-20
Genome
Hypo
Hyper
i
H3K9me3
Log2 converted H3K9me3 signals
p = 5.03E-16
p = 1.19E-79
p = 1.33E-79
j
H4K20me3
Processed CUT&RUN score
WT
W796A
-2.5 Kb
start
end
+2.5 Kb
peaks
k
L1Md_A chr3:78,336,081-78,345,303
WT
W796A
[0 - 24]
L1Md_A chr13:61,418,558-61,441,383
[0 - 25]
L1Md_T chr3:6,204,015-6,344,450
[0 - 46]

Supplementary Figure 5. eRRBS profiling of DNA methylation in 1KO-ESC cells reconstituted with either WT or BAH1-mutated form of DNMT1. (**a**) The rates of bisulfite conversion, labeled on top of columns, for all cytosines in each 1KO-ESC cell sample with expression of the indicated DNMT1, as determined by the unmethylated lambda DNA used as spike-in controls. TM denotes W464A/W465A/W796A. (**b, c**) Bar plots (b) and distribution plot (c) of absolute methylation levels for CpG sites with >5 coverage among 1KO-ESC lines with stable expression of the indicated DNMT1, as detected by eRRBS. (**d**) LC-MS analysis of global 5-mdC levels (calculated as 5-mdC/2MS analysis of g on the y axis) in 1KO-ESC lines after stable transduction of empty vector or the indicated DNMT1 (n=3 biological replicates). Data are mean±s.d. The two-tailed Student t-tests were performed to compare distributions between different groups. The data for 1KO and WT were adopted from those published previously[1]. (**e**) Distribution of weighted methylation scores at the H4K20me3 peaks categorized by quartile (Q1-Q4) of the descending H4K20me3 ChIP-seq signals. Scores are calculated after aggregating data from replicate samples per group. Peaks without mapped reads for a given sample group are ignored, resulting in N=6013 (WT Q1), N=6023 (W796A Q1), N=5908 (WT Q2), N=5910 (W796A Q2), N=5681 (WT Q3), N=5677 (W796A Q3), N=5409 (WT Q4), and N=5408 (W796A Q4). In the box-and-whisker plot, the box depicts the 25th to 75th percentiles, the black dot is the median, the whiskers extend to data points up to 1.5 IQR beyond the box, and open gray circles are data points outside the whisker range. (**f, g**) Distribution of weighted methylation scores at random genomic regions, which either lack H4K20me3 (f) or lack both H4K20me3 and H3K9me3 (g), which are size-matched to H4K20me3 peaks categorized by quartile. Scores are calculated after aggregating data from replicate samples per group. Random regions without mapped reads for a given sample group are ignored. For non-H4K20me3 randomized regions, N=5927 (WT Q1), N=5911 (W796A Q1), N=5836 (WT Q2), N=5840 (W796A Q2), N=5764 (WT Q3), N=5741 (W796A Q3), N=5588 (WT Q4), and N=5570 (W796A Q4). For random regions without H4K20me3 and H3K9me3, N=5918 (WT Q1), N=5906 (W796A Q1), N=5797 (WT Q2), N=5800 (W796A Q2), N=5709 (WT Q3), N=5718 (W796A Q3), N=5549 (WT Q4), and N=5532 (W796A Q4). In the box-and-whisker plot, the box depicts the 25th to 75th percentiles, the black dot is the median, the whiskers extend to data points up to 1.5 of IQR beyond the box, and open gray circles are data points outside the whisker range. (**h, i**) Bar plots showing the levels of H4K20me3 (h) or H3K9me3 (i) in ESCs, genome-wide (left) or within DMRs found to be associated with the W796A-mutated DNMT1 relative to WT, either hypo (middle) or hyper-methylated (right; for panels h-i, n = 8590 for Hypo, 4636 for Hyper and 8590 for randomized genome regions as a control). Y-axis, log2 converted ChIP-seq signals. DMR calling is determined by using methylkit. Box-and whisker plots depict 25-75% in the box, whiskers are 10-90%, and median is indicated. Statistical analysis in (e-i) used two-tailed Student's t-test. **(j)** Normalized H4K20me3 CUT&RUN signals over all called peaks in 1KO-ESCs rescued with exogeneous

DNMT1, wither WT or W796A-mutated. The start and end points of called H4K20me3 peaks are labeled. **(k)** IGV profiles of H4K20me3 CUT&RUN signals at the indicated retrotransposon elements (the LINE-1 class) in 1KO-ESCs rescued with exogeneous DNMT1, wither WT or W796A-mutated.

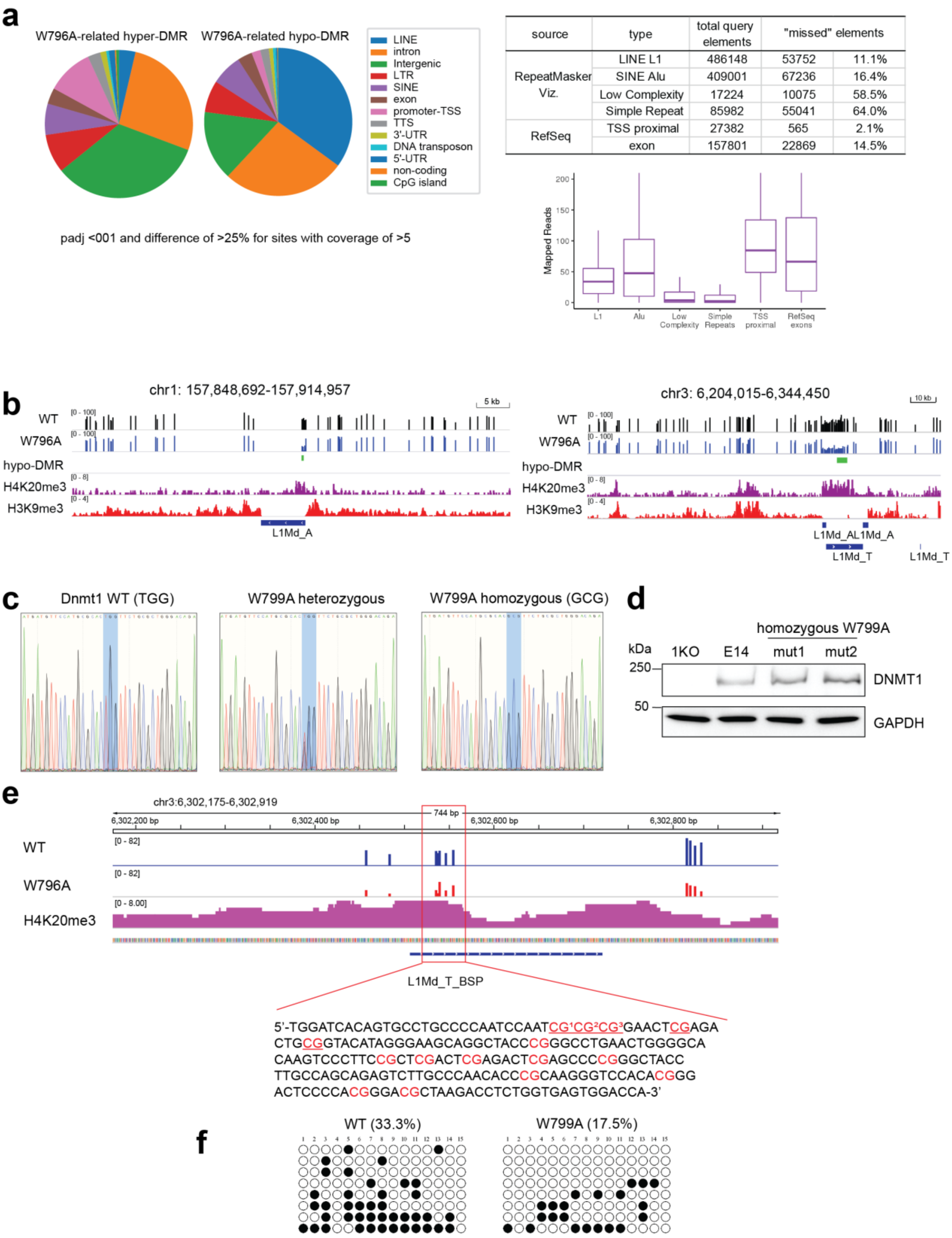

Supplementary Figure 6. Mutational analysis of DNMT1-mediated DNA methylation at LINE1. (**a**) Left panels: Pie chart showing distribution of the DMRs associated with the W796A-mutated DNMT1 relative to WT, either hypo (left) or

hyper-methylated (right), among the indicated genomic feature. DMR calling is determined by using methylkit. Right panels: a table for calculation of the fraction of “missed/ignored” sequencing reads (top) and boxplot showing the distribution of average mapped read depth (bottom) in each of the indicated group, including the repeated elements (LINE1/L1, SINE/Alu, simple repeat and low complexity repeats), exon and TSS-proximal (1kb upstream of annotated RefSeq TSS). eRRBS data analysis uses a cutoff of read depth of no less than 10 mapped read tags (per group), we define the ignored/missed elements as those that do not have mapped read depth of at least 10 at any coordinate in the element. Box-and whisker plots depict 25-75% in the box, whiskers are 10-90%, and median is indicated. P-value is calculated using logistic regression and then adjusted to padj value using sliding linear model (SLIM) method. (**b**) Representative IGV views show CpG methylation levels at the called W796A-asociated hypo-DMRs (green bar), either located in the chromosome 1 or 3, among 1KO-ESC lines with stable expression of WT (black) or the BAH1-mutated (blue) DNMT1. Cytosines covered by at least 5 reads according to eRRBS data are shown, with each site designated by a vertical line. The bottom panels show IGV view of H4K20me3 (purple) and H3K9me3 (red) in WT ESCs, and position of LINE-1. **(c)** Representative Sanger sequencing results verified the Dnmt1 W799A knock-in mutation, either heterozygous or homozygous, in the E14 ESCs. **(d)** Immunoblotting for Dnmt1 in Dnmt1-null ESCs (1KO), WT E14 ESCs, and those with homozygous Dnmt1 W799A mutation. The experiment was repeated 3 times with consistent results. (**e**) IGV views of eRRBS and H4K20me3 profiles at the indicated LINE-1 element L1Md_T (top), as well as the amplicon sequence (bottom) covering 15 of the highlighted CpG sites as examined by individual site-targeted bisulfite sequencing. (**f**) Site-targeted bisulfite sequencing results showed overall DNA methylation levels at a LINE-1 element, L1Md_T, in E14 ESCs with either WT Dnmt1 or knockin of homozygous Dnmt1 W799A mutations.

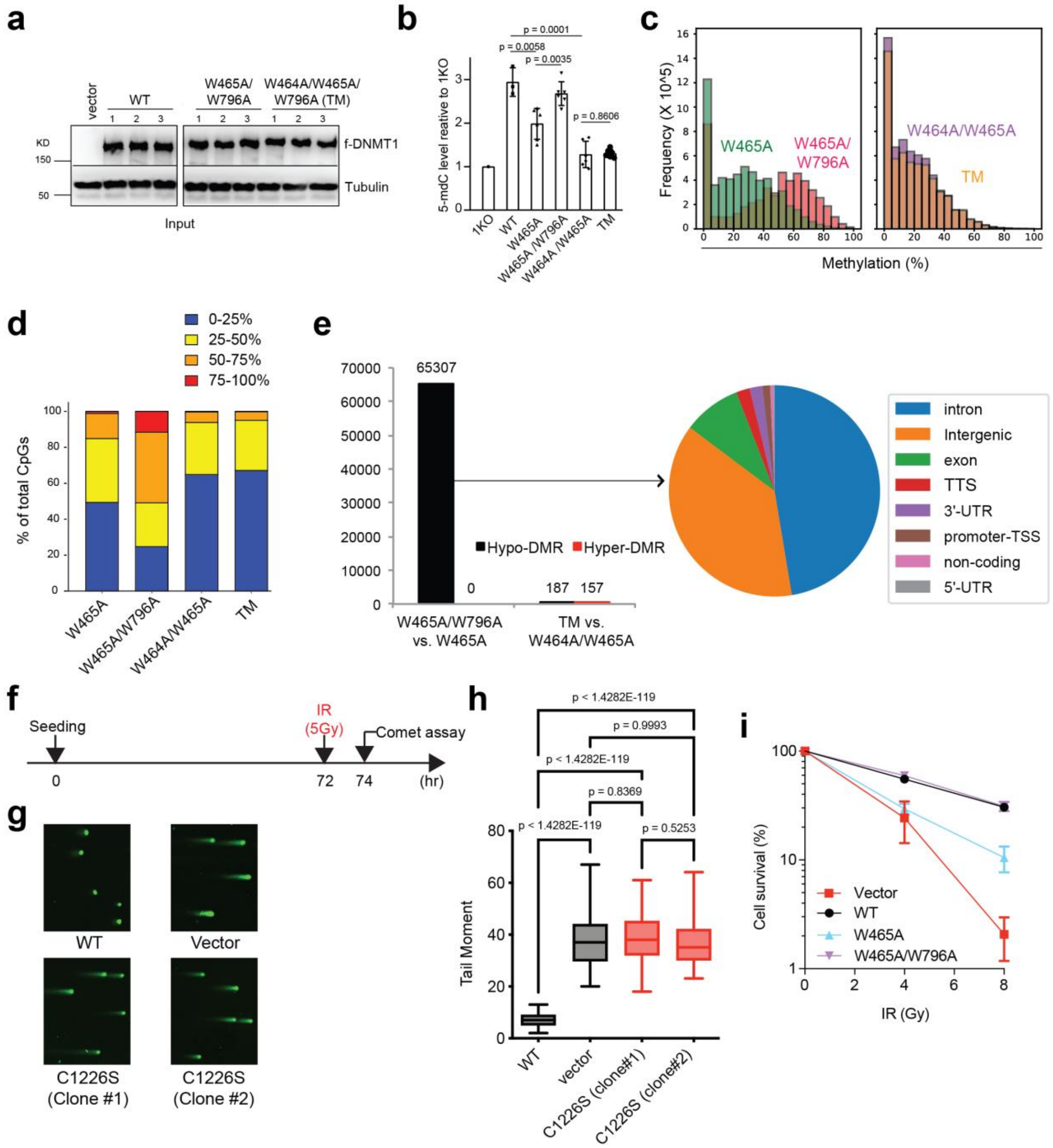

Supplementary Figure 7. DNMT1 BAH1 mutation crosstalks with the RFTS mutations in CpG methylation and genome stabilization. (**a**) Immunoblots of the indicated Flag-tagged DNMT1 after stable reconstitution into the independently derived 1KO-ESC lines. The experiment was repeated 3 times with consistent results. (**b**) LC-MS analysis of global 5-methyl-2-deoxycytidine (5-mdC) levels (calculated as 5-mdC/2-deoxyguanosine on the y-axis) in 1KO-ESC lines after stable transduction of empty vector or the indicated DNMT1 (n = 3 biological replicates for 1KO and WT; n = 6 biological replicates for W465A, W465A/W796A, W465A/W796A and TM). Data are mean ± s.d. Statistical analysis used two-tailed Student's t-test. The data for 1KO, WT, W465A and W464A/W465A were adopted from those published previously[1]. (**c, d**)

The distribution (**c**) and bar plots (**d**) showing absolute methylation levels of CpG sites with >5 coverage among 1KO-ESC lines with stable expression of the indicated DNMT1, as detected by eRRBS. (**e**) Summary of the total number of DMRs (left), either hyper-DMRs or hypo-DMRs, and pie chart (right) showing genomic distribution of the DMRs associated with BAH1 mutation (W796A) when introduced to the indicated RFTS mutant (either W465A or W464A/W465A) in 1KO-ESC cells. TM denotes W464A/W465A/W796A mutation. (**f**) Experimental scheme of the comet assay for (g,h). (**g**) Representative images of comet assay of DNMT1-/- 1KO-ESC cells complemented with empty vector or DNMT1, either WT or a catalytically dead mutant (C1226S; two different clones #1, and #2). (**h**) Box plot showing the tail moment of 1KO-ESC cells complemented with vector (n=103), WT (n=106), or C1242S (clone #1, n=110; clone #2, n=129). Each bar on the plot indicates the max value, upper quartile, median value, lower quartile, and minimum values, respectively. Plot was created and analyzed using one-way ANOVA with post Tukey analysis. (**i**) Survival of 1KO-ESC cells, reconstituted with vector control or the indicated DNMT1, post-treatment of IR. Data are mean ± s.d. (n = 3 biologically independent replicates).

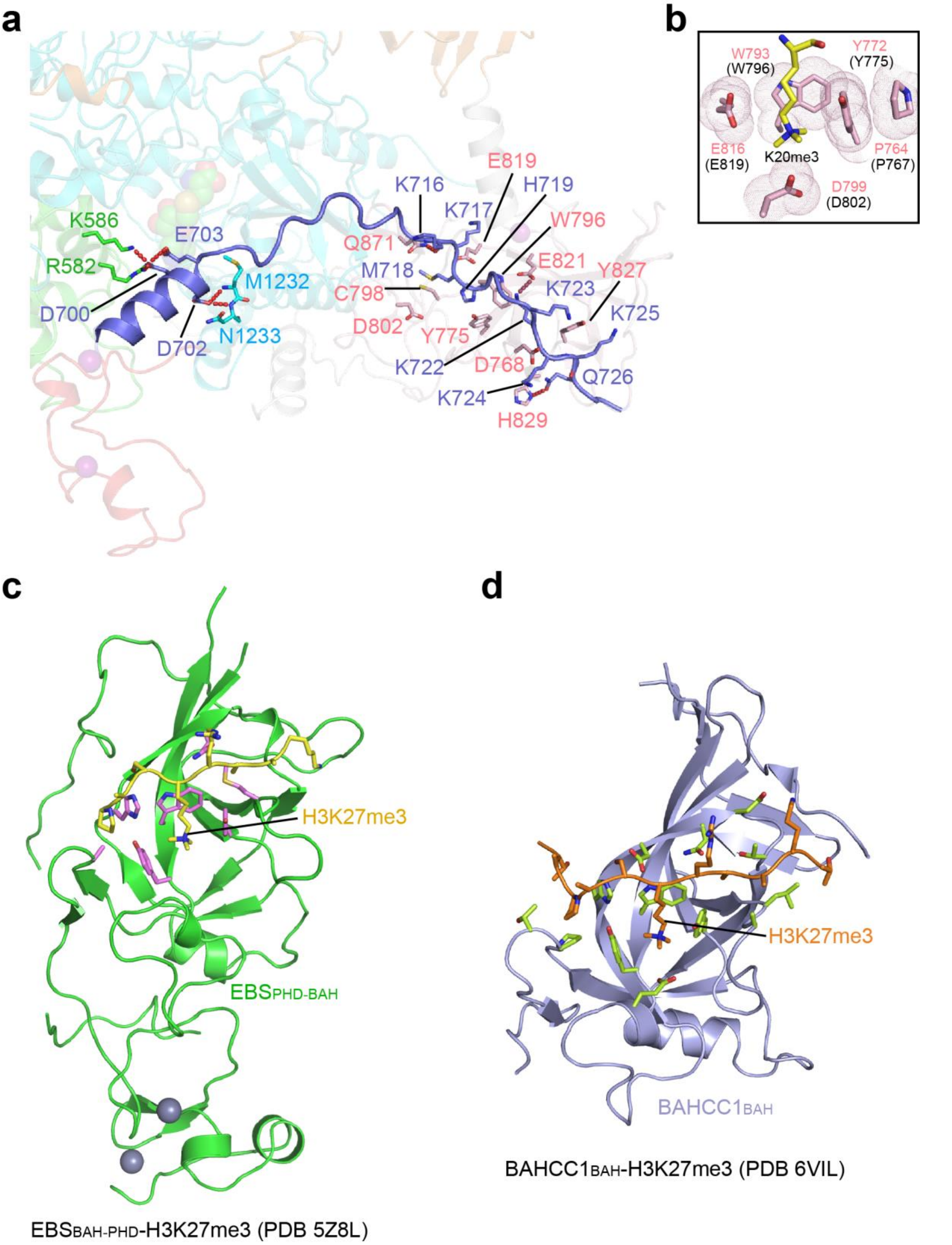

Supplementary Figure 8. Evolutionarily conserved histone recognition by the BAH domains. (**a**) Close-up view of the autoinhibitory linker-mediated intramolecular interaction of DNMT1. The autoinhibitory linker (slate) forms contacts with the BAH1 domain (light pink), as well as the RFTS (green) and MTase (cyan) domains. The

interacting residues are shown in stick representation. The hydrogen bonds are depicted as dashed lines. (**b**) The H4K20me3-binding pocket of $bDNMT1_{BAH1}$, with the equivalent pocket residues in $hDNMT1_{BAH1}$ labelled in parentheses. (**c**) Ribbon representation of the crystal structure of plant EBS BAH-PHD ($EBS_{BAH-PHD}$) dual domain bound to a H3K27me3 peptide (PDB 5Z8L). The H3K27me3-binding sites (magenta) are shown in stick representation. (**d**) Ribbon representation of the crystal structure of mouse BAHCC1 BAH ($BAHCC1_{BAH}$) domain bound to a H3K27me3 peptide (PDB 6VIL). The H3K27me3-binding sites (limon) are shown in stick representation.

Supplementary Table 1. Summary of ITC binding parameters.

Protein	Peptide	K_d (μM)	N value
$hDNMT1_{BAH1}$, WT	H4(14-25)#	18.8 ± 2.6	0.75 ±0.16
$hDNMT1_{BAH1}$, WT	H4(14-25)K20me#	11.4 ± 2.5	0.74 ± 0.16
$hDNMT1_{BAH1}$, WT	H4(14-25)K20me2#	4.1 ± 1.1	0.74 ± 0.05
$hDNMT1_{BAH1}$, WT	H4(14-25)K20me3#	2.7 ± 0.6	0.79 ± 0.01
$hDNMT1_{BAH1}$, D768A	H4(14-25)K20me3#	11.5 ± 2.1	0.82 ± 0.26
$hDNMT1_{BAH1}$, Y775A	H4(14-25)K20me3	361 ± 17.0	1.00*
$hDNMT1_{BAH1}$, W796A	H4(14-25)K20me3	NDB	
$hDNMT1_{BAH1}$, E819A	H4(14-25)K20me3	9.4 ± 0.7	0.73 ± 0.01
$hDNMT1_{BAH1}$, E821A	H4(14-25)K20me3	91.7 ± 12.4	0.88 ± 0.06
$hDNMT1_{BAH1}$, D768A	H4(14-25)K20me2	9.3 ± 0.1	0.92 ± 0.01
$hDNMT1_{BAH1}$, Y775A	H4(14-25)K20me2	NDB	
$hDNMT1_{BAH1}$, W796A	H4(14-25)K20me2	NDB	
$hDNMT1_{BAH1}$, E819A	H4(14-25)K20me2	12.3 ± 1.0	0.87 ± 0.02
$hDNMT1_{BAH1}$, E821A	H4(14-25)K20me2#	35.4 ± 2.2	0.83 ± 0.03
$hDNMT1_{BAH1}$, WT	hDNMT1(714-727)	70 ± 10	1.00 ± 0.12
$hDNMT1_{BAH1}$, W796A	hDNMT1(714-727)	NDB	
$hDNMT1_{BAH1}$, Mut^{YW}	hDNMT1(714-727)	NDB	
$hDNMT1_{RFTS}$, WT+H4K20me3 (Mixed in 1:2 molar ratio)	H3(1-22)K9me3	NDB	
MBP	H4(14-25)K20me3	NDB	
MBP-$hDNMT1_{351-1600}$, WT	H4(14-25)K20me3	>300	
MBP-$hDNMT1_{351-1600}$, W796A	H4(14-25)K20me3	NDB	
MBP-$hDNMT1_{351-1600}$, Mut^{YW}	H4(14-25)K20me3	NDB	
MBP-$hDNMT1_{351-1600}$, Mut^{Linker}	H4(14-25)K20me3#	122.5 ± 3.3	1.45 ± 0.26
$JMJD2A_{TD}$	H4(14-25)K20me3#	0.51 ± 0.04	0.97 ± 0.04
$ORC1_{BAH}$	H4(14-25)K20me2#	8.7 ± 0.2	0.75 ± 0.01

NDB, no detectable binding. *N value was fixed to 1.00 for curve fitting. #The mean value and S.D. were derived from at least two-independent measurements. The rest were derived from single measurements.

Supplementary Table 2. List of Primers used in this study.

Gene_primer	Sequence	Species	Application
**CRISPR/Cas9-sgRNA**			
crRNA_W796A	AGATGATGTTCCATGCGCAC	mouse	CRISPR/Cas9-based editing and generation of a Dnmt1 W799A mutation
genotyping_Dnmt1_W796A_for	GCTCCATCGCTTGCTTTCAG	mouse	PCR genotyping for Dnmt1 W799A mutation in the E14 knockin model
genotyping_Dnmt1_W796A_rev	CCTTGCGTACTCCTGGTTGT		
W796A_ssODN	A*C*T*CGCCCACCAGGAACAGTTCCAGGGGGTCGGAGGTGGCTCCCAGGACTGTGTCTGTCCCAGCGCAGAACGCGTGCGCATGGAACATCATCTGACCATTTTTGTCTTCCCACAGAGCTGTGACCCTGGCTAGATAGAGT*G*G*T (*: phosphorothioate bonds)	mouse	CRISPR/Cas9-based editing and generation of a Dnmt1 W799A mutation
Dnmt1-ScF1	TCTAGCCAGGTATGCATGCC	mouse	Sequencing primer for genotyping of mouse Dnmt1 W799A mutation
Dnmt1-ScR1	TGGGACAGAACATAGGGACG		
**Biochemical Assay for protein interaction and enzymatic activity**			
DNMT1_728_PRSF_F	CGCGGCGGTCTCGGATCCAAGAATCGCATCTCTTGGG	human	Cloning of His-SUMO-tagged hDNMT1 BAH1 domain
DNMT1_900_PRSF_R	CGCGGCGGTCTCCTCGAGTTAAGCCAGACGGGCACAGCTCACACAG		
DNMT1_D768A_F	TTATTCCAGCTGATTCCTCAAAACCGCTGTAT	human	hDNMT1 BAH1 D768A mutation
DNMT1_D768A_R	AGGAATCAGCTGGAATAACAGAGACACAGTCC		
DNMT1_Y775A_F	AACCGCTGGCTCTAGCAAGGGTCACGGCGCTG	human	hDNMT1 BAH1 Y775A mutation
DNMT1_Y775A_R	CTTGCTAGAGCCAGCGGTTTTGAGGAATCATC		
DNMT1_W796A_F	ACGCCCACGCGTTCTGCGCTGGGACAGACACA	human	hDNMT1 BAH1 W796A mutation
DNMT1_W796A_R	GCAGAACGCGTGGGCGTGAAACATCTGCCCGT		
DNMT1_E819A_F	TGGTGGATGCATGTGAGGACATGCAGCTTTCA	human	hDNMT1 BAH1 E819A mutation
DNMT1_E819A_R	TCCTCACATGCATCCACCAAGAACA		

	GCTCCAG		
DNMT1_E821A_F	GATGAATGTGCGGACATGCAGCTT TCATATAT	human	hDNMT1 BAH1 E821A mutation
DNMT1_E821A_R	TGCATGTCCGCACATTCATCCACCA AGAACAG		
DNMT1_728_GST _F	GGGCCCCTGGGATCCAAGAATCGC ATCTCTTGGGTCGG	human	Cloning of GST-tagged hDNMT1 (728-1600)
DNMT1_1600_GS T_R	GTGGTGGTGCTCGAGTTAGGCTTT GGCCAACATACAAAGC		
hDNMT1-BAH1-F WD	CGATCGAATTCAAGAATCGCATCTC TTGGGTCGG	human	Cloning of GST-tagged hDNMT1 BAH1
hDNMT1-BAH1-RE V	CGATCGTCGACTTAAGCCAGACGG GCA		
Bovine_DNMT1_72 5_F	CCGCCGGATCCAAGAATCGGATCT CTT GGGTTGG	bovine	Cloning of His-SUMO-tagged bDNMT1 BAH1 domain.
Bovine_DNMT1_89 7_R	CCGCCCTCGAGTTAGGCCAGACGT GCACAGCTTGCGC		
DNMT1_MBP_F	GTACATCCAAGGATCCCCCAAGTG CATTCAGTGCGGGC	human	Cloning of His-MBP-tagged hDNMT1(351-1600).
DNMT1_MBP_R	GTCCTACAGGCGCGCCTCATTAGG CTTTGGCCAACATACAAAGC		
DNMT1_Linker_1F	GCGGCGCAGGGGAAGGCGGCGGC ACAGAACAAGAATCGCATC	human	hDNMT1 M718A/H719A/K723A K724A/K725A mutation
DNMT1_Linker_1R	GCCGCCGCCTTCCCCTGCGCCGCT TTTTTGGGTGACGGCATCTC		
DNMT1_Linker_2F	GGCGGCGGCAGCGAACAAGAATC GCATCTCTTGGG	human	hDNMT1 M718A/H719A/K723A/K7 24A/K725A/Q726A mutation
DNMT1_Linker_2R	GATTCTTGTTCGCTGCCGCCGCCTT CCCCTGCGCCGC		
hDNMT1_BAH2_M BP_F	GTACATCCAAGGATCCGAGATGAG GCAAAAAGAAATCC	Human	Cloning of His-MBP-tagged hDNMT1 BAH2 domain
hDNMT1_BAH2_M BP_R	GTCCTACAGGCGCGCCTCATCCAG GGCTACGGGCATGGTTGGGA		
JMJD2A_PRSF_F	ACAGATTGGTGGATCCGGGGCCTT GCAAAGCATC	Human	Cloning of His-SUMO-tagged JMJD2A Tudor domain.
JMJD2A_PRSF_R	CTTTACCAGACTCGAGTTAGGGAA GCTCTTCATCCAGTGTG		

SmFRET Assay

DNMT_LPETG_60 9_F	GCAGACCATCAGGCATTCTCTGCC GGAGACCGGATAACTCGAGTCTGG TAAAGAAACCGC	human	Generation of His-SUMO-tagged hDNMT1 (351-609)-LPETG.
DNMT1_LPETG_6	TGCCTGATGGTCTGCCGCCTCGCC		

09_R	TGGGCTCGCCTCTGTCCCAGCGTG		
DNMT1_C616_F	ACCAGGGAGAAGGACAGGGGACC CTGCAAAGCCACCACCACCAAGC	human	Generation of His-SUMO-tagged hDNMT1 (351-639) T616C-LPETG.
DNMT1_640_R	TTATCCGGTCTCCGGCAGATCCTTT TCAATTTG		
LPETG_PRSF_F	CTGCCGGAGACCGGATAACTC	Human	Generation of His-SUMO-tagged hDNMT1 (351-639) T616C-LPETG
DNMT1_612_R	GTCCTTCTCCCTGGTAGAATGCCTG ATGGTCTGCCG		
DNMT1 C409S_C420S_F	GTGTACTGTAAGCACGGTCACCTG AGTCCCATCGACACCGGCCTCATC G	human	hDNMT1 RFTS C409S/C420S mutation
DNMT1 C409S_C420S_R	GTGCTTACAGTACACACTGAAGCTG GTCAGTTTGTGCTGGGGAAGC		
DNMT1 C580S_F	TCCTGACGCCCAGCATGCGGGACC TGATCAAG	human	hDNMT1 RFTS C580S mutation
DNMT1 C580S_R	GGTCCCGCATGCTGGGCGTCAGGA AGATGGGC		
DNMT1_646_F	GTACATCCAAGGATCCAACGCCTTT AAGCGCCGGC	human	Cloning of His-MBP-tagged hDNMT1 (646-1606)-LPETG
DNMT1_ 1606_LPETG_R	GTCCTACAGGCGCGCCTCATCCGG TCTCCGGCAGAGCTGAGGCACTCT CTCGGGC		
DNMT1 S570C_F	GAGAGTTATGACGAGGCCGGGGAC TGTGATGAGCAGCCCATCTTCCTG	human	hDNMT1 RFTS S570C mutation
DNMT1 S570C_R	CAGGAAGATGGGCTGCTCATCACA GTCCCCGGCCTCGTCATAACTCTC		

Supplementary Table 3. List of antibodies used for cellular analysis

Antibody targets	Vendor	Catalog #	Application (Antibody dilution)
Dnmt1 Antibody (H-12)	Santa Cruz Biotechnology	sc-271729	immunoblot (1:200)
GAPDH (14C10) Rabbit mAb	Cell Signaling	#2118	immunoblot (1:5000)
Anti-Histone H4 (mono methyl K20) antibody	Abcam	ab9051	immunoblot (1:3000)
Anti-Histone H4 (di methyl K20)	Abcam	ab9052	immunoblot (1:3000)
Anti-Histone H4 (tri methyl K20) antibody	Abcam	ab9053	immunoblot (1:3000), CUT&RUN
Histone H3 antibody	Abcam	ab1791	immunoblot (1:3000)
goat anti-mouse IgG HRP	Santa Cruz Biotechnology	sc-2005	immunoblot (1:5000)
goat anti-rabbit IgG HRP	Santa Cruz Biotechnology	sc-2004	immunoblot (1:5000)

References

1. Ren, W. *et al.* Direct readout of heterochromatic H3K9me3 regulates DNMT1-mediated maintenance DNA methylation. *Proc Natl Acad Sci U S A* **117**, 18439-18447 (2020).
