# Supplementary material for: DNMT1 reads heterochromatic H4K20me3 to reinforce LINE-1 DNA methylation
Source: Nat Commun. 2021 May 3;12:2490. doi: 10.1038/s41467-021-22665-4 (PMC8093215; doi:10.1038/s41467-021-22665-4)
Supplement: Supplementary file 2 — Reporting Summary [file 41467_2021_22665_MOESM2_ESM.pdf]

nature research

Corresponding author(s): Gang Greg Wang, Jikui Song

Last updated by author(s): Feb 5, 2021

Reporting Summary

Nature Research wishes to improve the reproducibility of the work that we publish. This form provides structure for consistency and transparency in reporting. For further information on Nature Research policies, see Authors & Referees and the Editorial Policy Checklist .

Statistics

For all statistical analyses, confirm that the following items are present in the figure legend, table legend, main text, or Methods section.

n/a	Confirmed	
☐	☒	The exact sample size (n) for each experimental group/condition, given as a discrete number and unit of measurement
☐	☒	A statement on whether measurements were taken from distinct samples or whether the same sample was measured repeatedly
☐	☒	The statistical test(s) used AND whether they are one- or two-sided<br>*Only common tests should be described solely by name; describe more complex techniques in the Methods section.*
☒	☐	A description of all covariates tested
☒	☐	A description of any assumptions or corrections, such as tests of normality and adjustment for multiple comparisons
☐	☒	A full description of the statistical parameters including central tendency (e.g. means) or other basic estimates (e.g. regression coefficient) AND variation (e.g. standard deviation) or associated estimates of uncertainty (e.g. confidence intervals)
☐	☒	For null hypothesis testing, the test statistic (e.g. F, t, r) with confidence intervals, effect sizes, degrees of freedom and P value noted<br>*Give P values as exact values whenever suitable.*
☒	☐	For Bayesian analysis, information on the choice of priors and Markov chain Monte Carlo settings
☒	☐	For hierarchical and complex designs, identification of the appropriate level for tests and full reporting of outcomes
☒	☐	Estimates of effect sizes (e.g. Cohen's d, Pearson's r), indicating how they were calculated

Our web collection on statistics for biologists contains articles on many of the points above.

Software and code

Policy information about availability of computer code

Data collection	X-ray diffraction data were collected using the standard data collection software from synchrotron beamline 24-ID-E, NE-CAT at Advanced Photo Source (APS) and BL92 at Stanford Synchrotron Radiation Lightsource (SSRL).
Data analysis	For structural study, the HKL2000, XDS, PHENIX v1.16_3549-000, Coot v0.8.9 and Pymol v0.99 softwares were used for data processing and analysis. For DMR detection, methylKit software package (v.1.8.1) was used. Custom scripts used for eRRBS analyses are available upon request. ChIP-seq data were analyzed using BEDTools v2.24.0. For neutral comet assays, images for neutral comet assays were analyzed using Image J (v.1.53), and statistics and graph were calculated using Prism module of the GradPad software (v6).

For manuscripts utilizing custom algorithms or software that are central to the research but not yet described in published literature, software must be made available to editors/reviewers. We strongly encourage code deposition in a community repository (e.g. GitHub). See the Nature Research guidelines for submitting code & software for further information.

Data

Policy information about availability of data

All manuscripts must include a data availability statement. This statement should provide the following information, where applicable:

- Accession codes, unique identifiers, or web links for publicly available datasets
- A list of figures that have associated raw data
- A description of any restrictions on data availability

Coordinates and structure factors for the bDNMT1 BAH1-H4(14-25)K20me3 and bDNMT1 BAH1-H4(14-25)K20me2 complexes have been deposited in the Protein Data Bank under accession codes 7LML and 7LMM, respectively. The eRRBS data have been deposited in NCBI Gene Expression Omnibus under accession code GSE145698.

Field-specific reporting

Please select the one below that is the best fit for your research. If you are not sure, read the appropriate sections before making your selection.

☒ Life sciences ☐ Behavioural & social sciences ☐ Ecological, evolutionary & environmental sciences

For a reference copy of the document with all sections, see nature.com/documents/nr-reporting-summary-flat.pdf

Life sciences study design

All studies must disclose on these points even when the disclosure is negative.

Sample size	Biochemical and enzymatic assays were completed using wild type or mutants of DNMT1 fragments. eRRBS assays were completed using wild type or mutant DNMT1 plasmids. The sample size is sufficient to delineate the mutational effects of DNMT1.
Data exclusions	No data exclusion.
Replication	For in vitro DNA methylation assays, three independent measurements were performed for each sample and stated in figure legends. For cellular and genomics assays, at least 3 biological replicates were used and stated in figure legends. Data are presented as the mean ± SD of at least two independent experiments. Statistical analysis was performed with Student's t test for comparing two sets of data with assumed normal distribution. A p value of less than 0.05 was considered to be significant. All attempts at replication were successful.
Randomization	The assays performed in this study require a rational approach for activity comparison. Therefore, randomization is not applicable to our experimental set up.
Blinding	Blinding is not applicable to any biochemical or cellular assay performed in this study.

Reporting for specific materials, systems and methods

We require information from authors about some types of materials, experimental systems and methods used in many studies. Here, indicate whether each material, system or method listed is relevant to your study. If you are not sure if a list item applies to your research, read the appropriate section before selecting a response.

Materials & experimental systems

n/a	Involved in the study	
☐	☒	Antibodies
☐	☒	Eukaryotic cell lines
☒	☐	Palaeontology
☒	☐	Animals and other organisms
☒	☐	Human research participants
☒	☐	Clinical data

Methods

n/a	Involved in the study	
☒	☐	ChIP-seq
☒	☐	Flow cytometry
☒	☐	MRI-based neuroimaging

Antibodies

Antibodies used	Antibodies used for immunoblotting include α-Flag (Sigma; M2), Dnmt1 Antibody (H-12) (Santa Cruz Biotechnology sc-271729), GAPDH (14C10) Rabbit mAb (Cell Signalling #2118), H4K20me3 (Abcam ab9053), H3K9me3 (Abcam ab8898), general histone H3 (Abcam ab1791) and α-Tubulin (Sigma T5168). Antibodies used for histone peptide microarray and pulldowns include anti-GST (EpiCypher, 13-0022), anti-Rabbit-HRP (GE, NA934V), anti-GST (custom rabbit polyclonal, Gozani Lab), and anti-Rabbit-HRP antibodies (Cell Signaling Technologies, #7074).
Validation	Anti-Dnmt1 Antibody (H-12) is a mouse monoclonal IgG1 κ Dnmt1 antibody. Anti-Dnmt1 Antibody (H-12) is recommended for detection of Dnmt1 of mouse, rat and human origin by WB, IP, IF, IHC(P) and ELISA (See https://www.scbt.com/p/dnmt1-antibody-h-12?gclid=CjwKCAiA9vOABhBfEiwATCi7GBgvuFm0_QFBACYUo3j_AcPT_Fm94Y8XBj7-hsWSdNghaAqCgDr6rBoCc94QAvD_BwE).<br><br>anti-GST (custom rabbit polyclonal, Gozani Lab): This antibody has been validated in a published study (PMID 26439302)<br><br>GAPDH (14C10) Rabbit mAb detects endogenous levels of total GAPDH protein (https://www.cellsignal.com/products/primary-antibodies/gapdh-14c10-rabbit-mab/2118).<br><br>Anti-GST (EpiCypher, 13-0022) is used to label a GST-tagged fusion protein bound to specific peptides on the EpiTitan Histone Peptide Array (Catalog No. 11-2001). The GST antibody was then detected using an AlexaFluor 647-conjugated secondary antibody (https://www.epicypher.com/content/documents/tds/13-0022.pdf).

Anti-histone H3 recognizes total histone H3 independently of whether it's modified or not, making it an ideal control for nuclear loading and normalization of histone modifications levels (https://www.abcam.com/epigenetics/advantages-of-our-anti-histone-h3-antibody).

Anti-H3K9me3: Histone H3 (tri methyl K9) antibody (ab8898) is specific for Histone H3 tri methyl Lysine 9. Shows slight cross-reactivity with tri methyl K27, which shares a similar epitope (please see Western blot image). Does not react with mono or di methylated K9 (https://www.abcam.com/histone-h3-tri-methyl-k9-antibody-chip-grade-ab8898.html).

Anti-H4K20me3: Anti-Histone H4 (tri methyl K20) antibody - ChIP Grade. Purified using Sulpholink column with specific peptide linked via its cysteine residue (https://www.biocompare.com/9776-Antibodies/106587-Histone-H4-tri-methyl-K20-antibody-ChIP-Grade/).

Anti-FLAG M2: affinity purified monoclonal antibody binds to fusion proteins containing a FLAG peptide sequence. The antibody recognizes the FLAG peptide sequence at the N-terminus, Met-N-terminus,C-terminus, and internal sites of the fusion protein. (https://www.sigmaaldrich.com/catalog/product/sigma/f1804?lang=en&region=US)

α-Tubulin recognizes an epitope located at the C-terminal end of the α-tubulin isoform in a variety of organisms. It has been used in immunofluorescence Analysis, estern blotting/ Immunoblotting and for immunolabelling cells in electron microscopy. (See https://www.sigmaaldrich.com/catalog/product/sigma/t5168?lang=en&region=US)

Eukaryotic cell lines

Policy information about cell lines

Cell line source(s)	Dnmt1-knockout mouse embryonic stem cells (1KO-ESCs; a gift from Dr. M. Okano) were cultivated.
Authentication	Authentication of cell line identity, including that of parental and derived lines, was ensured by Tissue Culture Facility affiliated to the Lineberger Comprehensive Cancer Center of UNC at Chapel Hill using the genetic signature profiling and fingerprinting analysis.
Mycoplasma contamination	Every 1-2 month, a routine examination of cell lines in culture for any possible mycoplasma contamination was carried out using MycoAlert Mycoplasma Detection Kit (Lonza). No mycoplasma contamination was identified.
Commonly misidentified lines (See ICLAC register)	No commonly misidentified cell lines were used in the study.
