# Supplementary material for: DNMT1 reads heterochromatic H4K20me3 to reinforce LINE-1 DNA methylation
Source: Nat Commun. 2021 May 3;12:2490. doi: 10.1038/s41467-021-22665-4 (PMC8093215; doi:10.1038/s41467-021-22665-4)
Supplement: Supplementary file 3 — Description of Additional Supplementary Files [file 41467_2021_22665_MOESM3_ESM.pdf]

Description of Additional Supplementary Files

File Name: Supplementary Data 1
Description: Summary of eRRBS data obtained from the mouse Dnmt1-/- (1KO) ESC cells reconstituted with DNMT1, either WT or the indicated mutant.

File Name: Supplementary Data 2
Description: Lists of DMRs, called by DSS, which show the significantly altered CpG methylation levels in 1KO-ESCs post-transduction of a BAH1-mutated (W796A) form of DNMT1 relative to WT controls. P-value is calculated using logistic regression and then adjusted to padj value using sliding linear model (SLIM) method.

File Name: Supplementary Data 3
Description: Lists of DMRs, called by methylkit, that show the significantly altered CpG methylation levels in 1KO-ESCs post-transduction of a BAH1-mutated (W796A) form of DNMT1 relative to WT controls. P-value is calculated using logistic regression and then adjusted to padj value using sliding linear model (SLIM) method.

File Name: Supplementary Data 4
Description: List of genomic coordinates of hypo-DMRs found in the BAH1-mutated versus WT control cells, which reveals enrichment of DMRs at 5'-UTR of L1 elements.
